# A Proposed New Species Complex within the Cosmopolitan Ring Nematode *Criconema annuliferum* (de Man, 1921) Micoletzky, 1925

**DOI:** 10.3390/plants11151977

**Published:** 2022-07-29

**Authors:** Ilenia Clavero-Camacho, Juan Emilio Palomares-Rius, Carolina Cantalapiedra-Navarrete, Pablo Castillo, Gracia Liébanas, Antonio Archidona-Yuste

**Affiliations:** 1 Institute for Sustainable Agriculture (IAS), Spanish National Research Council (CSIC), Avenida Menéndez Pidal s/n, Campus de Excelencia Internacional Agroalimentario, ceiA3, 14004 Córdoba, Spain; iclavero@ias.csic.es (I.C.-C.); palomaresje@ias.csic.es (J.E.P.-R.); ccantalapiedra@ias.csic.es (C.C.-N.); p.castillo@csic.es (P.C.); 2 Department of Animal Biology, Plant Biology and Ecology, University of Jaén, Campus ‘Las Lagunillas’ s/n, Edificio B3, 23071 Jaén, Spain; gtorres@ujaen.es; 3 Andalusian Institute of Agricultural and Fisheries Research and Training (IFAPA), Centro Alameda del Obispo, 14004 Córdoba, Spain

**Keywords:** cytochrome c oxidase c subunit 1 (COI), cryptic species, D2-D3 expansion domains of the large ribosomal subunit (28S), internal transcribed spacer (ITS), multivariate morphometric analysis, species delimitation

## Abstract

Ring nematodes are obligate ectoparasites on cultivated and wild herbaceous and woody plants, inhabiting many types of soil, but particularly sandy soils. This study explored the morphometrical and molecular diversity of ring nematodes resembling *Criconema annuliferum* in 222 soil samples from fruit crops in Spain, including almond, apricot, peach and plum, as well as populations from cultivated and wild olives, and common yew. Ring nematodes of the genus *Criconema* were detected in 12 samples from under *Prunus* spp. (5.5%), showing a low to moderate nematode soil densities in several localities from southeastern and northeastern Spain. The soil population densities of *Criconema* associated with *Prunus* spp. ranged from 1 nematode/500 cm^3^ of soil in apricot at Sástago (Zaragoza province) to 7950 and 42,491 nematodes/500 cm^3^ of soil in peach at Ricla and Calasparra (Murcia province), respectively. The integrative taxonomical analyses reveal the presence of two cryptic species identified using females, males (when available), and juveniles with detailed morphology, morphometry, and molecular markers (D2-D3, ITS, 18S, and COI), described herein as *Criconema paraannuliferum* sp. nov. and *Criconema plesioannuliferum* sp. nov. All molecular markers from each species were obtained from the same individuals, and these individuals were also used for morphological and morphometric analyses. *Criconema paraannuliferum* sp. nov. was found in a high soil density in two peach fields (7950 and 42,491 nematodes/500 cm^3^ of soil) showing the possibility of being pathogenic in some circumstances.

## 1. Introduction

Plant-parasitic nematodes of the family Criconematidae Taylor, 1936 [1] received the common name of ‘ring nematodes’ because of the body cuticle shows wide and prominent annuli. Ring nematodes are extensively, albeit not uniformly, distributed throughout the world [2]. They are obligate ectoparasites on cultivated and wild herbaceous and woody plants, inhabiting many types of soil but particularly sandy soils [3]. Several species of ring nematodes have been reported as parasitic and important pest of crops, causing damage to roots [4,5], but experimental evidence about the potential damage for crops induced by some species of criconematids is still lacking [6]. The genus *Criconema* Hofmänner & Menzel, 1914 [7] comprises about 100 nominal species characterized by body annuli smooth or with crenate margins, but without appendages in females, submedian lobes absent but six pseudolips projecting in the first lip annuli, stylet strong (mean 76 µm, but ranging from 37 to 159 µm) [2]. The slightly retrorse annuli found only in *Criconema* are special adaptations for helping to move slowly along soil particles keeping a straight body [2,6]. The taxonomy and systematic of *Criconema* has undergone great modifications over several decades [2,6]. The genus *Criconema* was proposed by Hofmänner & Menzel [7] to accommodate *Criconema guernei* (Certes) Menzel in Hofmänner & Menzel [7] and *Criconemoides morgense* (Hofmänner in Hofmänner & Menzel, 1914) [7]. De Grisse and Loof [8] proposed the genus *Nothocriconema* for species with modified lip annuli lacking submedian lobes, a closed vulva overhung by its anterior lip and juveniles with longitudinal rows of scales or spines. However, Andrássy [9,10] synonymized *Lobocriconema* with *Nothocriconema* and considered *Criconema* as a genus dubium [10], and Ebsary (1981) reinstated *Lobocriconema* and proposed two new genera, *Nothocriconemella* and *Paracriconema*, for ‘two morphologically distinct groups of species included in *Nothocriconema*’ [11]. Raski and Luc [12] re-established this genus as valid, gave a combination of morphological characters to separate *Criconema* from the closest genera (viz. presence of cuticular scales or spines in juveniles, the absence of such ornamentation in adult females, and the differentiation of the lip annuli, usually larger and thicker than the body annuli), and considered *Amphisbaenema*, *Nothocriconema, Lobocriconema, Nothocriconemella, Notholetus*, and *Paracriconema* as junior synonyms of *Criconema*. Finally, Siddiqi [6] proposed several subgenera within *Criconema* (*Amphisbaenema*, *Criconema*, *Nothocriconema* and *Notholetus*), whereas *Paracriconema* was considered as a synonym of *Criconema* [6].

The genus *Criconema* displays a great phenotypic plasticity and absence of clear diagnostic characters, and for this reason, molecular taxonomy and DNA barcoding is providing accurate and useful tools for species identification in recent years [13,14,15,16,17]. Subbotin et al. [13] analysed the phylogenetic relationships of the main lineages recognised in Criconematidae based on D2-D3 expansion segments of the 28S nuclear ribosomal RNA, and their data support the monophyly of *Criconema*. Although Subbotin et al. [13] suggested that additional studies were needed to recognise those characters more informative from a phylogenetical point of view.

The ring nematode *Criconema annuliferum* is widely distributed in several European countries, including Austria, Belgium, Bulgaria Netherlands, Poland, Russia, Slovak Republic, Spain [16,18,19,20,21,22,23], but also in Africa [24], Asia [2,25], New Zealand [26], and South America [27]. In Spain, it has been widely reported in several localities including cultivated and natural environments, such as, olive, peach, pepper, wild vegetation, and forests [20,28,29,30,31,32,33,34,35,36,37,38]. Although Gómez-Barcina et al. [32] and Escuer et al. [20] provided descriptions, measurements, and illustrations, including SEM pictures, of specimens from several Spanish populations of *C. annuliferum*, no molecular data on these populations are available. In view of this, a new and extensive nematode survey in agricultural (with special focus on *Prunus* plantations) and natural areas was conducted to update and thus clarify the distribution and occurrence of this species in Spain. In fact, we detected eleven unidentified populations of *Criconema* which, based on detailed morphological and morphometric observations by light microscopy, appeared indistinguishable from the morphology of *C. annuliferum*. This prompted us to undertake exhaustive multivariate and genetic analyses with previous reported data including described populations of *C. annuliferum*. A detailed integrative approach was conducted to clarify the taxonomic status of these ring nematode populations, where preliminary results indicated that these populations appeared to be undescribed species and thus, the existence of a new species complex within the genus *Criconema*. 

The main objectives of this study were to (i) accurately identify with morphological and morphometrical approaches the new populations of *Criconema* detected in an extensive nematode survey on *Prunus* plantations compared with other in natural habitats and agricultural systems such as cultivated olive trees in Spain; (ii) discover the diversity of *C. annuliferum*-species complex through integrative taxonomy, combining morphological analysis and a species delineation approach based on multivariate analysis of morphometric data and genetic methods; (iii) describe new species of the genus *Criconema* belonging to the *C. annuliferum*-complex group; (iv) provide molecular characterization of these *Criconema* populations using ribosomal (D2-D3 expansion segments of 28S rRNA, Internal Transcribed Spacer region (ITS) rRNA) and the mitochondrial cytochrome c oxidase subunit 1 (COI); (iv) study phylogenetic relationships within *Criconema* spp. using the obtained molecular markers.

## 2. Results

**Figure 1:**
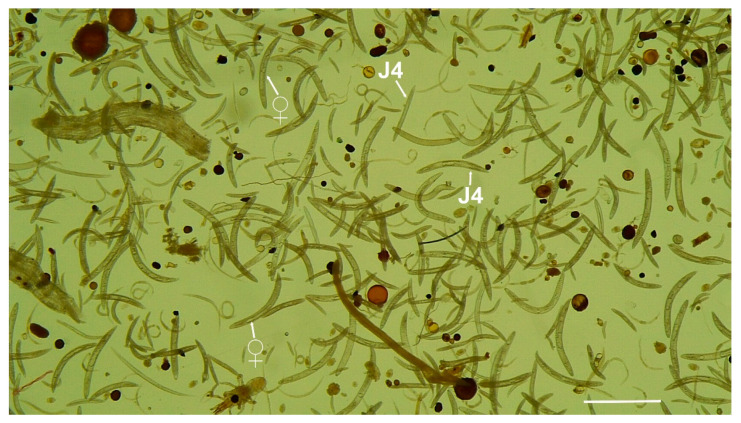
Soil sample extraction (500 cm^3^) from peach in Calasparra, Murcia, Spain showing numerous female and juveniles (arrowed) of *Criconema paraannuliferum* sp. nov. “♀” = female, and “J4” = fourth-stage juvenile. (Scale bar = 500 µm).

From the 219 analysed samples from under *Prunus* spp. in Spain, ring nematodes of the genus *Criconema* were detected in 12 (5.5%), including fruit crops, such as almond, apricot, peach and plum, showing a low to moderate soil nematode density in several localities of southeastern and northeastern Spain (Table 1). The population densities of *Criconema* in soil under *Prunus* spp. ranged from 1 nematode/500 cm^3^ of soil under apricot at Sástago (Zaragoza province) to 7950 and 42,491 nematodes/500 cm^3^ of soil (Figure 1) under peach at Ricla and Calasparra (Murcia province), respectively, and all of them were identified under integrative taxonomy as a new cryptic species, herein described as *Criconema paraannuliferum* sp. nov. Specimens of *Criconema* with a close morphological resemblance to *Criconema paraannuliferum* sp. nov. from under *Prunus* spp. were collected from soil under common yew, wild and cultivated olives simultaneously in several localities of Jaén and Cádiz provinces (southern Spain). These samples showed population densities from 12 to 37 specimens/500 cm^3^ of soil. Identification of specimens of *Criconema* from soil below wild and cultivated olives resulted in species identical to those found in soil below *Prunus* spp., whereas two species morphologically closely related were detected in the soil sample below common yew, (about 50% each in soil numbers), but clearly separated by molecular and morphometric analyses, and described herein as *Criconema paraannuliferum* sp. nov. and *Criconema plesioannuliferum* sp. nov. (Table 1).

### 2.1. Species Delimitation Using Morphometry by Principal Component Analysis

**Figure 2:**
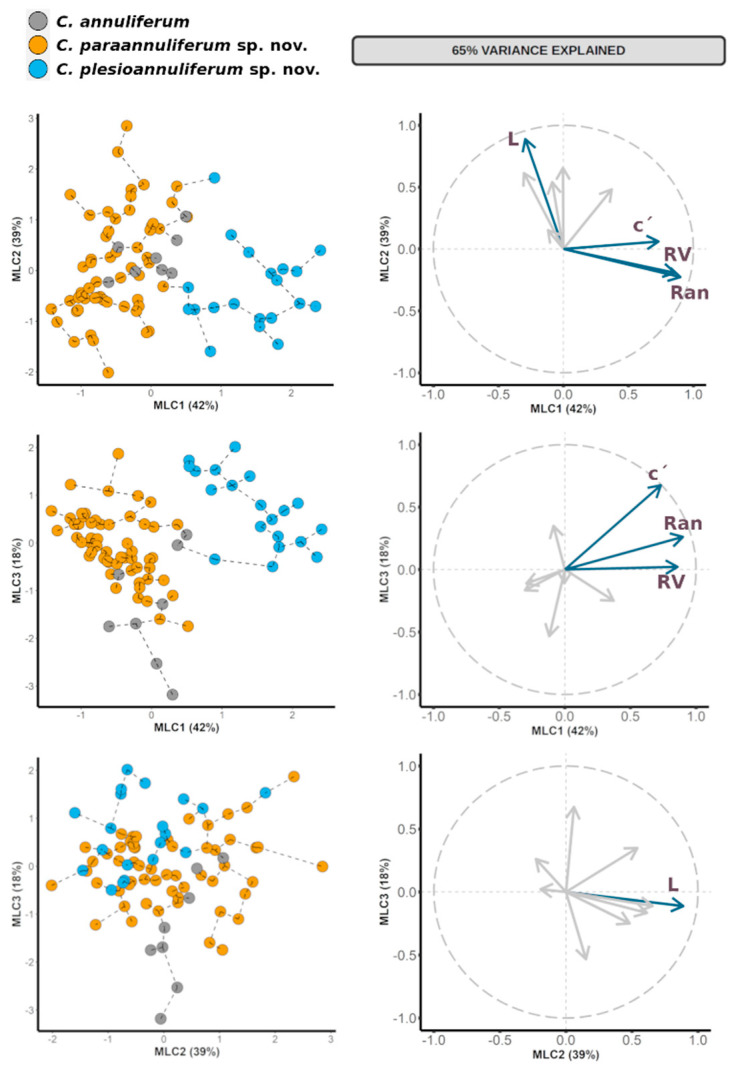
Principal component on morphometric characters to characterize *Criconema annuliferum*-complex species with a superimposed minimum spanning tree (based on Euclidean distance). Blue arrows indicate morphometric variables with projected eigenvalue > 0.70.

In the maximum likelihood factor analysis (FA), the first three components (sum of squares (SS) loadings > 1) accounted for 65% of the total variance in the morphometric characteristics of the *C. annuliferum*-complex (Table 2). The eigenvalues for each character were used to interpret the biological meaning of the factors. First, the maximum likelihood component 1 (MLC1) was dominated by number of annuli between the posterior end of the body and the vulva (RV), number of annuli on the tail (Ran) and for the c’ ratio with high positive correlations (eigenvalues = 0.86, 0.90 and 0.73, respectively). This component was, therefore, related with the shape and size of the posterior part of nematode and tail. The ML2 was dominated by a high positive correlation for the nematode body length (L) (eigenvalue = 0.89), thereby relating this component to the overall nematode size. Finally, the ML3 was dominated by a high positive correlation for the c’ ratio (eigenvalue = 0.67). This component was then related with tail shape. Overall, these results suggest that all the extracted components were related to the overall size and shape of nematodes in each population, especially the size and shape of the posterior part of the nematode. The results of the factor analyses were represented graphically in Cartesian plots where specimens from populations of the *C. annuliferum*-complex were projected on the plane of the x- and y-axes, respectively, as pairwise combinations of components 1 to 3 (Figure 2). The specimens of the *C. annuliferum*-complex of all species were projected in the graphic representation showing an expanded distribution along the plane for all the projected combinations of the components owing to their wide morphometric variation within species and/or populations. This was more pronounced for *C. paraannuliferum* sp. nov. and *C. annuliferum*, where a high number of populations were considered [16,19,27,39,40]. Consequently, we did not detect a clear separation among *C. paraannuliferum* sp. nov. and *C. annuliferum*, with all the specimens belonging to these species being projected at random for all the projected combinations. However, and except for the projection on the plane of MLC2 and MLC3, where specimens of all species were randomly plotted, we observed that most specimens belonging to *C. plesioannuliferum* sp. nov. were spatially separated from the rest of species within the *C. annuliferum*-complex (Figure 2). This spatial distribution was dominated by MLC1 accounting for 27% of the total of variance (42% of the proportion explained). This spatial separation was mainly dominated by MLC1 grouped species according to the number of annuli between posterior end of body and vulva (RV), number of annuli on tail (Ran) and for the c’ ratio (Table 2, Figure 2). Thus, specimens of *C. plesioannuliferum* sp. nov. having a higher number of annuli between the posterior end of the body and the vulva (RV), higher number of annuli on the tail (Ran) and higher values for the c’ ratio were located at the right side, and on the opposite side were *C. paraannuliferum* sp. nov., while those of *C. annuliferum* were characterized by lower values for these diagnostic characters (Figure 2) [16,27,39,40]. However, specimens of *C. paraannuliferum* sp. nov. and *C. annuliferum* having similar values for RV, Ran and the c’ ratio were grouped among them, not showing spatial separation between the species (Figure 2). A minimum spanning tree (MST) superimposed on the plot of the first three principal components showed the same patterns observed with factor analysis. That is, a separation of *C. plesioannuliferum* sp. nov. but no clear separation between *C. paraannuliferum* sp. nov. and *C. annuliferum* within the *C. annuliferum*-complex (Figure 2). 

### 2.2. Species Delimitation Based on Ribosomal and Mitochondrial DNA

Species delimitation by ribosomal and mitochondrial DNA was based on four statistical parameters: intra-/inter-species variation, P ID (Liberal) values, posterior probability of clades on Bayesian analyses, and Rosenberg’s P_AB_ (Table 3). Analyses of species delimitation demonstrated that *C. annuliferum* from Belgium was clearly separated from *C. paraannuliferum* sp. nov. and *C. plesioannuliferum* sp. nov. The intra- and inter-species molecular variation for D2-D3 region of all three species was higher than 0.10, except for *C. plesioannuliferum* that was 0.09 (Table 3), suggesting that the probability of species identification with this gene is low. However, the variation for the ITS and COI genes were clearly below 0.10 (Table 3), suggesting that the probability of species identification with these loci is high [42]. Similarly, the P ID (Liberal) values for all three species and locus were ≥0.93 suggesting that species can be adequately delimited [43,44]. Furthermore, all clade supports for the three loci were strong (PP = 1.00), and the Rosenberg’s P_AB_ values also support the monophyly of each species separately [45].

### 2.3. Systematics

#### 2.3.1. *Criconema paraannuliferum* sp. nov.

**Figure 3:**
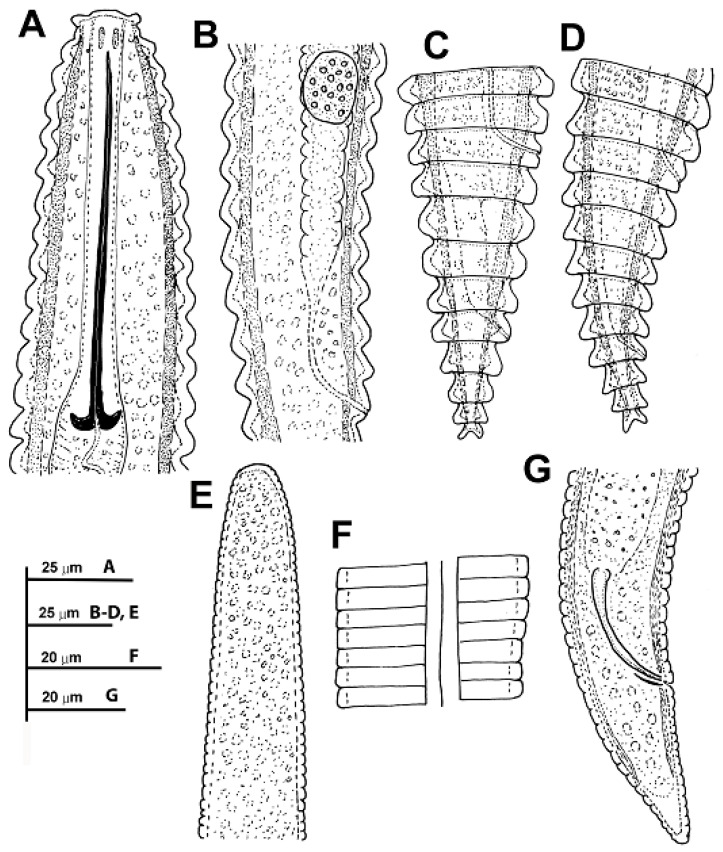
Line drawings of *Criconema paraannuliferum* sp. nov. (**A**), female anterior region; (**B**), detail of spermatheca; (**C**,**D**), female posterior region; (**E**), male anterior region showing absence of stylet; (**F**), male lateral field at mid-body; (**G**), male posterior region.

**Figure 4:**
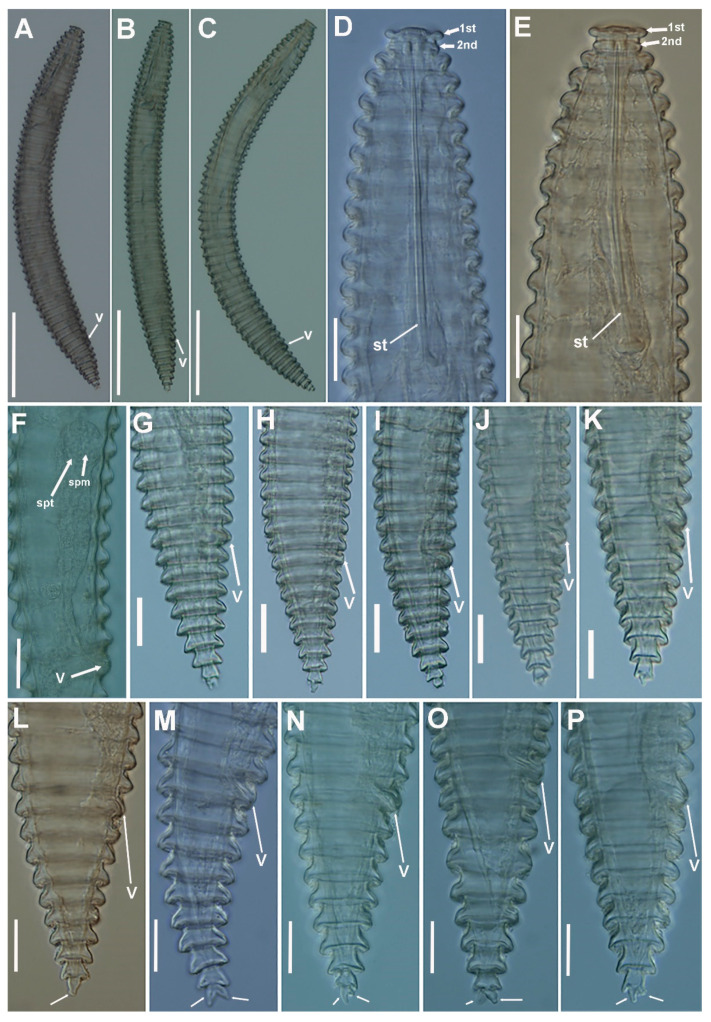
Light micrographs of *Criconema paraannuliferum* sp. nov. females. (**A**–**C**), whole female; (**D**,**E**), anterior region, 1st and 2nd body annuli (arrowed); (**F**), posterior region showing spermatheca filled with sperm (arrowed); (**G**–**P**), posterior region showing vulva (arrowed). Abbreviations: spt = spermatheca, spm = sperm; st = stylet; V = vulva; 1st, 2nd = first- and second-body annuli. Scale bars: 20 µm.

**Figure 5:**
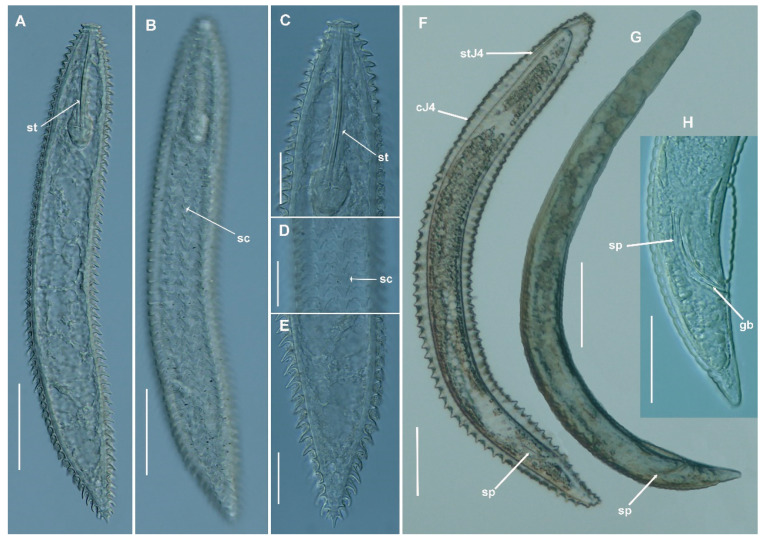
Light micrographs of *Criconema paraannuliferum* sp. nov. fourth-stage juveniles (J4) and males. (**A**,**B**), whole fourth-stage juvenile, showing stylet and rows of scales (arrowed); (**C**), J4 anterior region. (**D**), J4 mid-body portion showing rows of scales (arrowed); (**E**), J4 posterior region; (**F**), whole male enveloped by J4 cuticle. (**G**), whole male. (**H**), male tail showing spicules and gubernaculum (arrowed). Abbreviations: cJ4 = cuticle J4; gb = gubernaculum; sc = scales; sp = spicules; st = stylet; stJ4 = stylet of J4. Scale bars: (**A**,**B**) = 50 µm; (**C**–**E**,**H**) = 20 µm; (**F**,**G**) = 100 µm.

**Figure 6:**
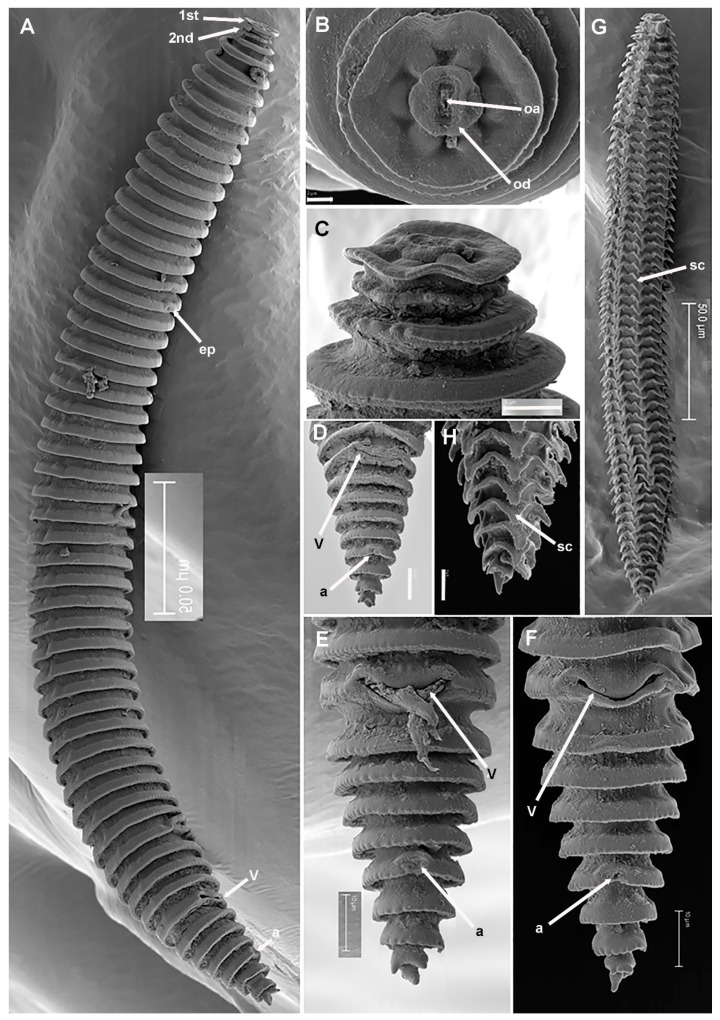
SEM micrographs of *Criconema paraannuliferum* sp. nov. female and fourth-juvenile stage. (**A**), whole female; (**B**), female in face view showing oral disc and oral aperture (arrowed); (**C**), detail of 1st and 2nd body annuli; (**D**–**F**), posterior region showing vulva and anus (arrowed); (**G**), whole juvenile showing files of scales; (**H**), posterior region showing files of scales (arrowed) and minute acute projections. Abbreviations: a = anus; ep = excretory pore; oa = oral aperture; od = oral disc; sc = scales; V = vulva; 1st, 2nd = first- and second-body annuli. Scale bars: (**A**,**G**) = 50 µm; (**B**) = 2 µm; (**C**) = 5 µm; (**D**–**F**,**H**) = 10 µm.

(Figure 3, Figure 4, Figure 5 and Figure 6, Table 4). http://zoobank.org/urn (accessed on 28 April 2022): lsid:zoobank.org:act: 226AA8E8-E597-4947-B262-11E83E64D827. 

##### Description

*Female*: Body ventrally arcuate after heat relaxation, body annuli thick 7.5–10.5 µm wide, rounded with smooth edges and without anastomoses. Lip region low, with two annuli, the first annulus wide, anteriorly directed, and the second annulus narrower and forming a collar-like appearance. The SEM photographs show an elongate oral aperture, surrounded by a rounded oral plate, without submedian lobes, and six distinct hexaradiate pseudolips. Stylet long, generally straight, but slightly curved in some specimens, representing 17.1–23.5% of body length or 57.4–78.3% of pharynx length, knobs anchor-shaped, 8.5–10.0 µm wide and 3.0–4.0 µm high. Dorsal pharyngeal gland opening 9.0 ± 1.0 (8.0–12.0) µm from base of stylet. Nerve ring surrounding isthmus, located at 92–129 µm from anterior end. Excretory pore usually 1–2 annuli before pharyngeal base, 104–154 µm from anterior end. Hemizonid not seen. Reproductive system monodelphic-prodelphic, outstretched (190–264 µm long), composed of a long ovary with oocytes arranged in one single row. Spermatheca not developed and without sperm in all the populations from *Prunus* spp., wild olive and common yew, but were well developed, rounded to slightly oval [14.6 ± 2.4 (12.0–18.0) µm, 14.0 ± 1.5 (12.0–15.5) µm] and filled with rounded sperm (ca. 1 µm wide) in all females of the population from cultivated olive from Castillo de Locubín with presence of males. Vulva closed, anterior lip not overhanging posterior one, vagina straight, vulva-anus distance 1.3–2.3 times of tail length. Anus hardly visible, tail conical, with 4–6 annuli, terminal annulus usually bi-lobed.

*Male*: Not found in any of the six populations collected from peach, wild olive, and common yew, but only detected in the population from cultivated olive from Castillo de Locubín, Jaén province. Not frequent (1 male: 30 females). Most males were detected inside the cuticle of fourth-stage juvenile. Body ventrally curved, tapering to posterior region. Lip region rounded, stylet absent, pharynx not developed, lateral fields with 3 incisures. Testis straight, 56 (47–62)% of total body length. Tail conoid with rounded terminus, bursa absent, spicules slender, cephalated and ventrally curved, gubernaculum simple, slightly curved ventrally.

*Juveniles*: Body similar to that of female. Lip region with collar-like first annulus, annuli margins with 8–10 rows of projections in all the annuli along body. Margins of projections with a row of 6–8 short bristles only distinguishable under SEM observations.

##### Diagnosis and Relationships

*Criconema paraannuliferum* sp. nov. is characterized by a medium-sized female body 396–657 µm, stylet = 85–113 µm, V = 82.8–89.8, c = 18.4–31.2, c’ = 1.0–1.5, R = 57–66, RV = 7–10, Ran = 3–6, VL/VB = 1.4–2.0, a conical tail with terminal annulus usually bi-lobed, and males only detected in one locality with bursa absent and spicules 30.5–37.0 µm.

According to the dichotomic key of species within *Criconema* by Geraert [6], the new species needs to be compared with species of *Criconema* sharing a group of characters including mid-body annuli smooth and tail annuli not ornamented, stylet 80–130 µm long, no distinct differentiation of the lateral field, anterior vulval lip overhanging or overlapping posterior lip, RV = 8–19, R = 53–76, last tail annuli in line with the general slope of the tail, and the first lip annulus wider than following lip annuli [6]. The new species is morphologically very similar to *C. annuliferum*, and resembles *Criconema crotaloides* (Cobb, 1924) Schuurmans-Stekhoven & Teunissen, 1938 [46,47] and *Criconema iranicum* Azimi & Pedram, 2020 [48]. The morphological and morphometrical data from *C*. *paraannuliferum* sp. nov. for the three populations collected in soils from below peach, one in common yew, and two in cultivated and wild olives during this study are within the ranges of the original description of *C. annuliferum*, as well as those of Peneva et al. [40] from oak forests in Russia, and those of Etongwe et al. [16] from Belgium, and these minor differences are within the range of intraspecific variation. In addition, the new species can be separated from *C. annuliferum* by spicule length (30.5–37.0 vs. 51–53 µm). However, molecular characterization of the Belgian populations by ribosomal and mitochondrial genes clearly separated *C. annuliferum* from *C. paraannuliferum* sp. nov. in this study. The new species can be differentiated from *C. crotaloides* by body length (396–657 vs. 517–820 µm), RV (7–10 annuli from terminus vs. 11–14), and Ran (3–6 annuli from terminus vs. 6–9) [19,49]. Finally, it can be separated from *C. iranicum* by RV (7–10 vs. 9–11), and longer stylet (85–113 vs. 76.5–84.0 µm) [48].

##### Molecular Characterization

Twenty gene sequences from the D2-D3 region of the 28S rRNA (ON705053–ON705072), twenty-two ITS (ON705081–ON705102), fourteen 18S rRNA (ON705034–ON705047), and thirty-six from COI (ON648825–ON648860) were generated for this new species. Overall, intraspecific variation was 1 nucleotide for D2-D3, 99.4% similarity (0–4 nucleotides difference) for ITS, no variation for 18S rRNA, and 97.1% similarity (0–22 nucleotides difference) for COI. The highest COI variability within each population of this species was found in wild olive at Prado del Rey, Cádiz province (differing from 0 to 12 nucleotides), while no variability was detected within common yew population at Valdepeñas, Jaén province. The closest species to *C. paraannuliferum* sp. nov. was *C. plesioannuliferum* sp. nov., being 97.9–98.0% similar for the D2-D3 region (ON705073–ON705080) (differing by 13 nucleotides), and 93.5–94.7% similar to *C. annuliferum* (MN783697–MN783702) (differing by 37–42 nucleotides), and 93.8% similar (differing by 43 nucleotides and 2 indels) to *C. demani* (MN628432). The ITS region was 91.5 to 92.1% like *C. plesioannuliferum* sp. nov. (ON705103–ON705116) and 84.5–85.9% like *C. silvum* (MF683236–MF683237) (differing in 47 to 56 nucleotides, 86 to 103 nucleotides, 10 to 14 indels, 27 to 34 indels), respectively. The 18S rRNA showed high similarity values, being higher than 99% among all *Criconema* spp. deposited in the GenBank, and specifically, 99.5 to 99.4% like *C. plesioannuliferum* sp. nov. (ON705048–ON705051) and *C*. *crotaloides* (HM116022) respectively. For the COI gene sequences (ON648825–ON648860), the similarity values were 92.0 and 93.1% (differing in 41 to 55 nucleotides and no indels) from *C. plesioannuliferum* sp. nov., 89–90%) (differing in 37 to 40 nucleotides and 0 indel) from *C. annuliferum* (MN782387–MN782395) and 89–90% (differing in 62 to 75 nucleotides and 0 indel) from *C*. *crotaloides* (MN710680–MN710699). All molecular markers studied, except for 18S rRNA, clearly separated the new species from other species of *Criconema*. Unfortunately, no molecular data for *C. iranicum* were found in GenBank. Because two closely related species were detected in the soil sample from common yew, the few male specimens were confirmed molecularly (100% similarity) to belong to *C. paraannuliferum* sp. nov. 

##### Type Habitat and Locality

*Criconema paraannuliferum* sp. nov. was found in the rhizosphere of peach (coordinates 38°12′21.3″ N, 1°42′23.9″ W); the municipal district of Calasparra, Murcia province, southeastern Spain. Additional localities and host plants are reported in Table 1.

##### Etymology

The species epithet, *paraannuliferum*, refers to Gr. prep. para, alongside of and resembling, N.L. masc. n. *annuliferum*, because of its close resemblance to *Criconema annuliferum*.

##### Type Material

Holotype female (PR-129-06), 20 paratypes females, 4 fourth-stage juveniles paratypes (slide numbers PR-129-01 to PR129-05, PR129-08 to PR129-11) were deposited in the Nematode Collection of the Institute for Sustainable Agriculture, CSIC, Córdoba, Spain; two females at Istituto per la Protezione delle Piante (IPP) of Consiglio Nazionale delle Ricerche (C.N.R.), Sezione di Bari, Bari, Italy (PR129-12); and two females deposited at the USDA Nematode Collection (slide T-7629p).

#### 2.3.2. *Criconema plesioannuliferum* sp. nov.

**Figure 7:**
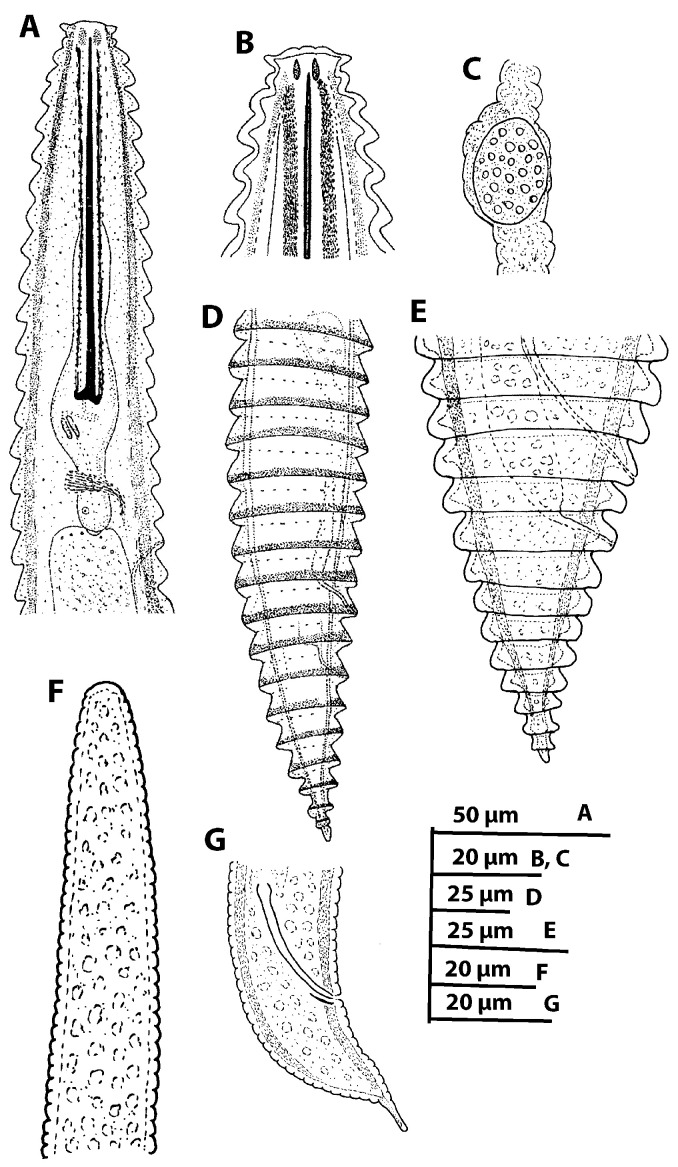
Line drawings of *Criconema plesioannuliferum* sp. nov. (**A**), female pharyngeal region; (**B**), detail of female lip region; (**C**), detail of spermatheca; (**D**,**E**), female posterior region; (**F**), male anterior region showing absence of stylet; (**G**), male posterior region.

**Figure 8:**
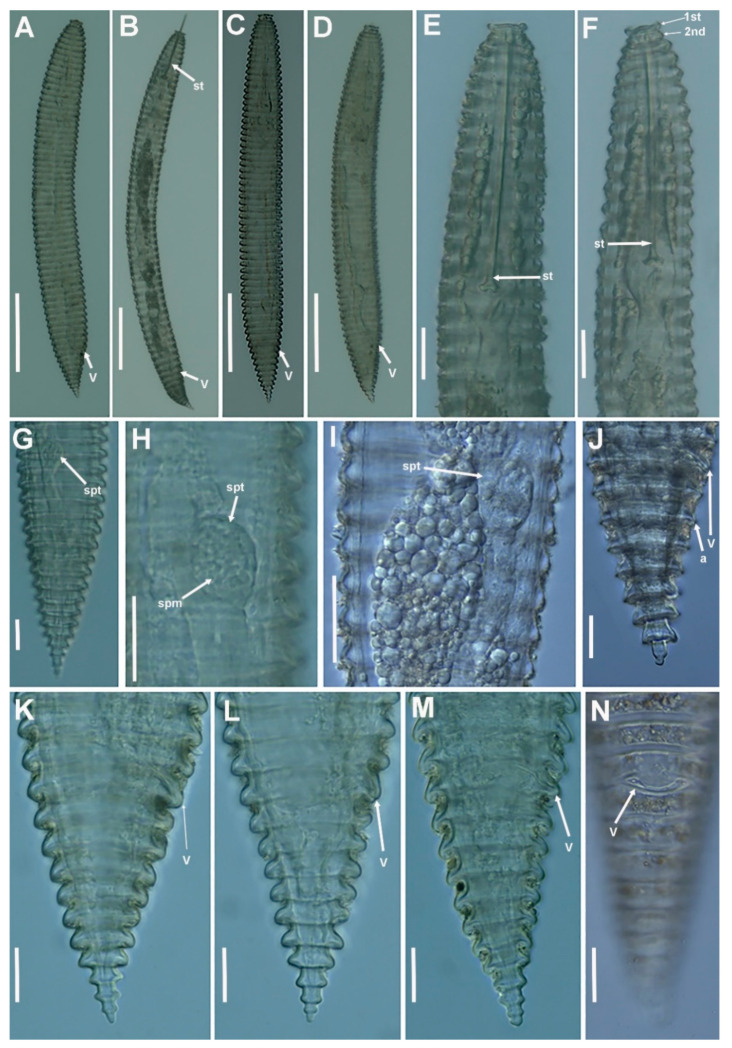
Light micrographs of *Criconema plesioannuliferum* sp. nov. females. (**A**–**D**), whole females (stylet and vulva arrowed); (**E**,**F**), anterior region, 1st and 2nd body annuli (arrowed); (**G**), posterior region with spermatheca (arrowed); (**H**,**I**), detail of spermatheca and sperm (arrowed); (**J**–**N**), posterior region showing vulva and anus (arrowed). Abbreviations: a = anus; spm = sperm; spt = spermatheca; st = stylet; V = vulva; 1st, 2nd = first- and second-body annuli. Scale bars: (**A**–**D**) = 100 µm; (**E**–**N**) = 20 µm.

**Figure 9:**
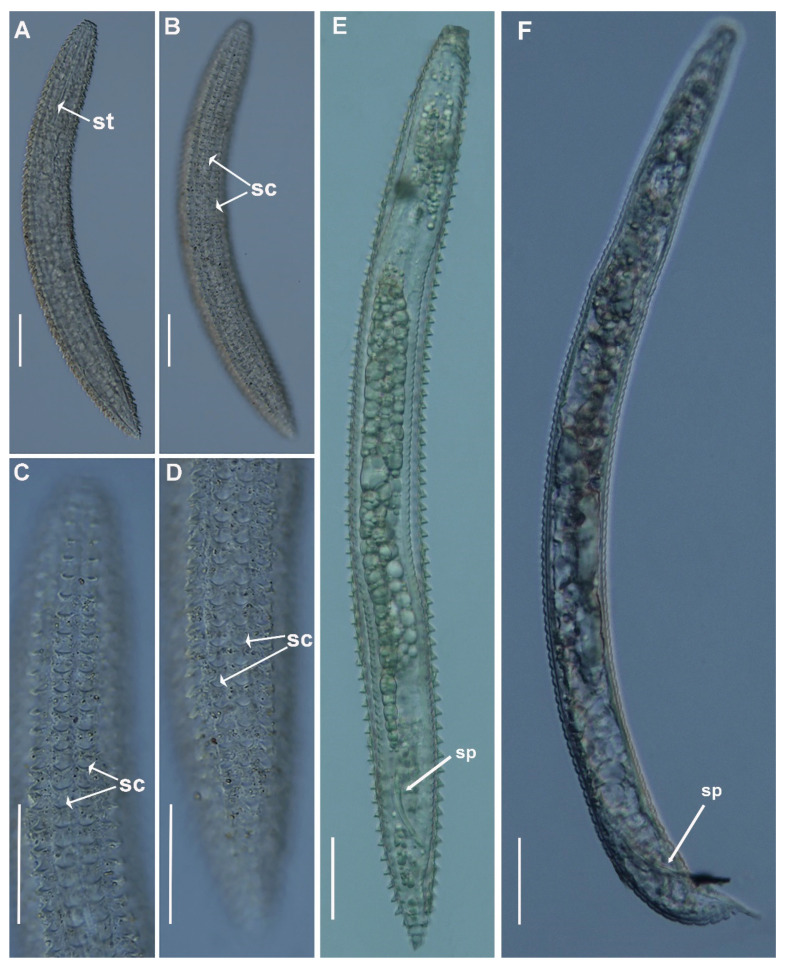
Light micrographs of *Criconema plesioannuliferum* sp. nov. juveniles and male. (**A**,**B**), whole juvenile, showing stylet and rows of scales (arrowed); (**C**), anterior region, showing rows of scales (arrowed); (**D**), posterior region showing rows of scales (arrowed); (**E**), whole male included in fourth-stage cuticle, showing spicules (arrowed); (**F**), whole male showing spicules (arrowed). Abbreviations: sc = scales; sp = spicules; st = stylet. Scale bars: (**A**–**D**) = 50 µm, (**E**,**F**) = 30 µm.

**Figure 10:**
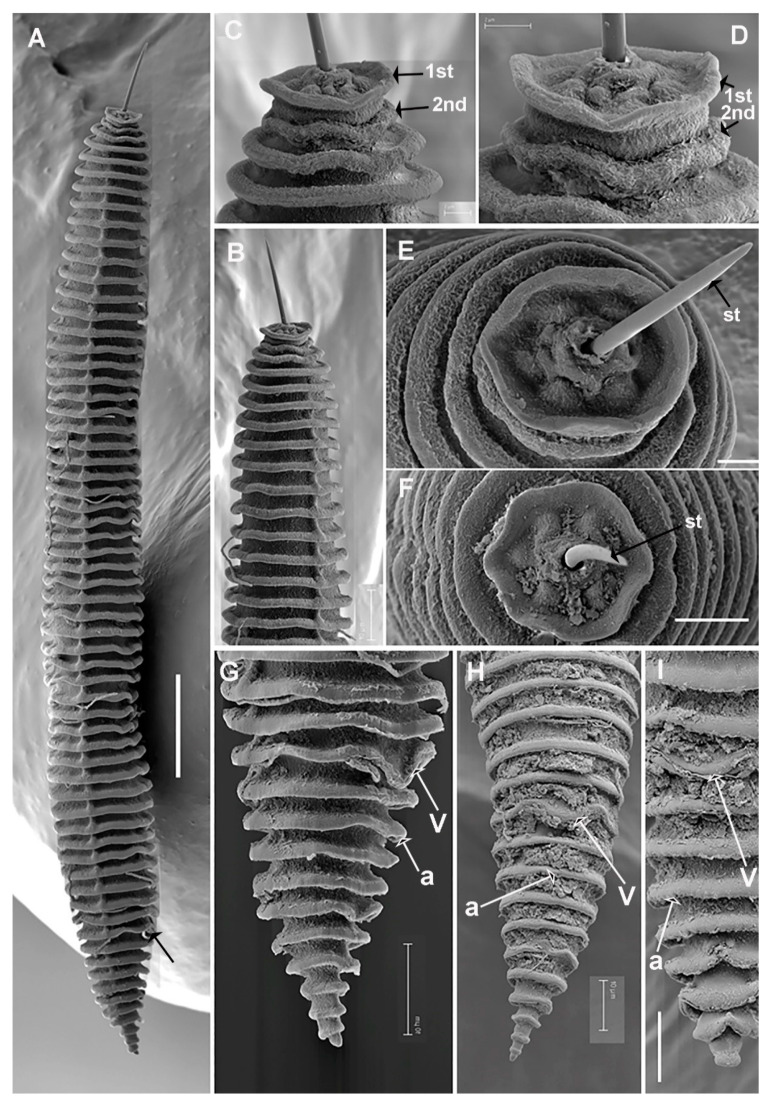
SEM micrographs of *Criconema plesioannuliferum* sp. nov. females. (**A**), whole female; (**B**), anterior region; (**C**,**D**), detail of 1st and 2nd body annuli; (**E**,**F**), in face view; (**G**–**I**), posterior region showing vulva and anus (arrowed). Abbreviations: a = anus; st = stylet; V = vulva; 1st, 2nd = first- and second-body annuli. Scale bars: (**A**) = 25 µm; (**B**) = 10 µm; (**C**–**E**) = 2 µm; (**F**) = 5 µm; (**G**–**I**) = 10 µm.

**Figure 11:**
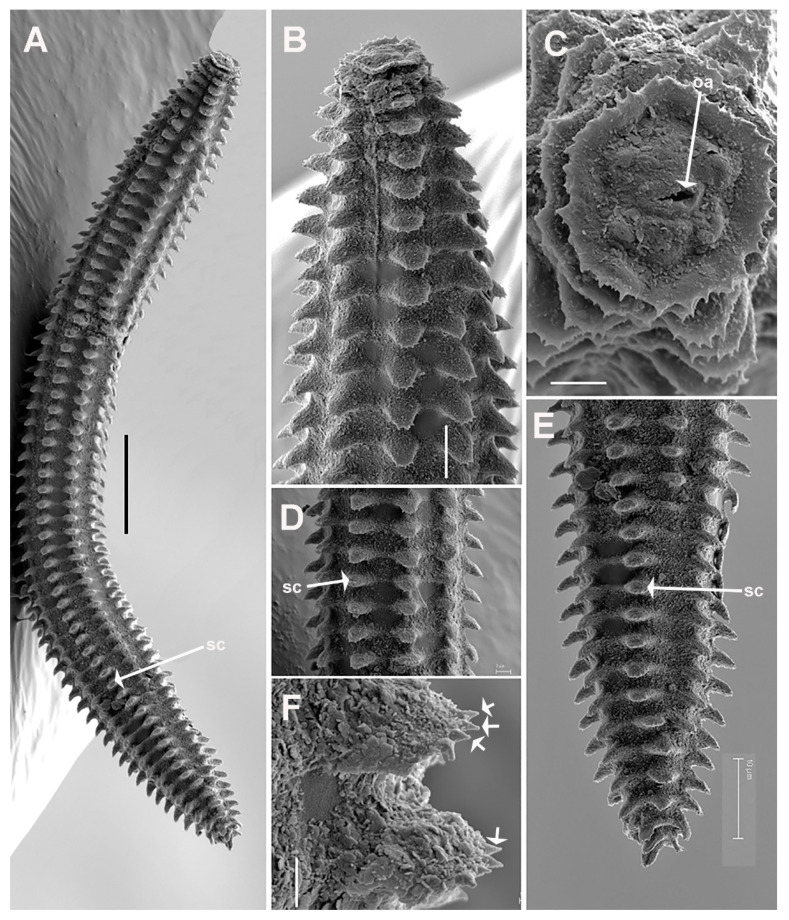
SEM micrographs of *Criconema plesioannuliferum* sp. nov. juveniles. (**A**), whole juvenile showing files of scales; (**B**), anterior region; (C), in face view; (**D**), mid-body region; (**E**), posterior region showing files of scales (arrowed); (**F**), detail of scales showing minute acute projections (arrowed). Abbreviations: oa = oral aperture; sc = scales. Scale bars: (**A**) = 25 µm; (**B**) = 5 µm; (**C**,**D**) = 2 µm; (**E**) = 10 µm; (**F**) = 1 µm.

(Figure 7, Figure 8, Figure 9, Figure 10 and Figure 11, Table 5). http://zoobank.org/urn (accessed on 28 April 2022): lsid:zoobank.org:act: 009C1AD3-595F-472A-9F27-80AC205F02A8. 

##### Description

*Female*: Body almost straight to slightly ventrally arcuate after heat relaxation, body annuli thick 8.0–12.0 µm wide, rounded with smooth edges and without anastomoses. Lip region low, with two annuli, the first annulus wide, anteriorly directed, and the second annulus narrower and forming a collar-like appearance. The SEM photographs show a rounded oral aperture, surrounded by a rounded oral plate, without submedian lobes, and six distinct hexaradiate pseudolips. The SEM photographs show a type of rudimentary cord joining the body annuli from the 5th annulus to the caudal region (Figure 10). Stylet long, generally straight but slightly curved in some specimens, representing 16.1–24.7% of body length or 50.2–89.3% of pharynx length, knobs anchor-shaped, 7.5–10.0 µm wide and 3.0–4.0 µm high. Dorsal pharyngeal gland opening 10.0 ± 2.4 (8.0–18.0) µm from base of stylet. Nerve ring surrounding isthmus, located at 70–155 µm from anterior end. Excretory pore usually 0–1 annulus behind pharyngeal base, 105–198 µm from anterior end. Hemizonid not seen. Reproductive system monodelphic-prodelphic, outstretched (159–354 µm long), composed of a long ovary with oocytes arranged in one single row, spermatheca rounded to oval (10–12 × 14–20 µm), filled with rounded sperm (1.0–1.5 µm wide). Vulva closed, anterior lip not overhanging posterior one, vagina straight, vulva-anus distance 0.3–0.5 times of tail length. Tail abruptly narrows to a conical shape, with 8–10 annuli, and finely pointed terminus, and last annulus not lobed.

*Male*: Not common (1 male: 7 females). Body ventrally curved, tapering to posterior region. Bursa absent, spicules slender, cephalated and ventrally curved, gubernaculum simple, slightly curved ventrally. Lip region rounded, stylet absent, pharynx not developed, lateral fields not detected. Testis straight, 40 (36–43)% of total body length. Tail conoid, ending in an abruptly narrow ventral peg.

*Juveniles*: Body like that of female. Lip region with collar-like first annulus, annuli margins with 8–10 rows of projections in all the annuli along body. Margins of projections with a row of 5–6 short bristles only distinguishable under SEM observations.

##### Diagnosis and Relationships

*Criconema plesioannuliferum* sp. nov. is characterized by a medium-sized body 372–658 µm, stylet = 86–108 µm, V = 81.7–88.5, c = 8.8–13.7, c’ = 1.3-1.8, R = 57–67, RV = 9–12, Ran = 8–10, VL/VB = 1.6–2.0, tail abruptly narrows to a conical shape, with 8–10 annuli, and finely pointed terminus, and last annulus not lobed, and males rare with bursa absent and spicules 29.0–32.0 µm.

According to the dichotomic key of species within *Criconema* by Geraert [6], the new species needs to be compared with species of *Criconema* sharing a group of characters including mid-body annuli smooth and tail annuli not ornamented, stylet 80–130 µm long, not distinct differentiation of the lateral field, anterior vulval lip overhanging or overlapping posterior lip, RV = 8–19, R = 53–76, last tail annuli in line with the general slope of the tail, and the first lip annulus wider than following lip annuli [6]. The new species is morphologically very similar to *C. annuliferum* and *C. paraannuliferum* sp. nov., and resembles *Criconema crotaloides* (Cobb, 1924) Schuurmans-Stekhoven & Teunissen, 1938 [46,47] and *Criconema iranicum* Azimi & Pedram, 2020 [48]. The morphological and morphometrical data of the population from common yew is within the ranges of *C. paraannuliferum* sp. nov. and *C. annuliferum*, except for ratios related with tail [16,40]. The new species can be separated from *C. paraannuliferum* sp. nov. by tail shape (abruptly narrowing to a conical shape, with 8–10 annuli, finely pointed terminus, and last annulus not lobed vs. conical with terminal annulus usually bi-lobed), RV (9–12 vs. 7–10 annuli from terminus), Ran (8–10 vs. 3–6 annuli from terminus), VL/VB (1.6–2.0 vs. 1.4–2.0), c ratio (8.8–13.7 vs. 18.4–31.2), c’ ratio (1.3–1.8 vs. 1.0–1.5). It can be differentiated from *C. crotaloides* by body length (372–658 vs. 517–820 µm), body annuli R (57–67 vs. 64–76), RV (9–12 vs. 11–14 annuli from terminus) and Ran (8–10 annuli from terminus vs. 7–9) [19,49]. Finally, it can be separated from *C. iranicum* by a longer stylet (86–108 vs. 76.5–84.0 µm), c ratio (8.8–13.7 vs. 19.8–31.4), c’ ratio (1.3–1.8 vs. 0.9–1.4) [48].

##### Molecular Characterization

Eight gene sequences D2–D3 of the 28S rRNA (ON705073–ON705080), fourteen ITS (ON705103–ON705116), four 18S rRNA (ON705048–ON705051), and twenty-four COI (ON648861–ON648884), were generated for this new species without intraspecific sequence variations, at exception of the COI gene, with one variable position. The closest species to *C. plesioannuliferum* sp. nov. were *C. paraannuliferum* sp. nov. and *C. annuliferum*, being 98.8 to 98.2%, 93.5 to 94.4% similar for the D2–D3 region (ON705053–ON705072, MN783697–MN783702) (differing in 12 to 13 nucleotides, 36 to 42 nucleotides, and 0 to 1 indel, 0 indel), respectively; unfortunately, no data was available for the D2–D3 region from *C. crotaloides* or *C. iranicum*. The ITS region was 91.5 to 92.1% similar to *C. paraannuliferum* sp. nov. (ON705081–ON705102) (differing in 47 to 56 nucleotides and 10 to 14 indels); no data were available from this region for the morphological related species *C. annuliferum*, *C. crotaloides* or *C. iranicum*. The 18S rRNA gene showed similarity values above to 99% with all *Criconema* sp. deposited in GenBank, including *C. paraannuliferum* sp. nov. (ON705034–ON705047) and *C. crotaloides* (HM116022). Finally, for the COI gene sequences (ON648861–ON648884), the similarity values were 92.0 and 93.1% (differing in 41 to 55 nucleotides and no indels) from *C. paraannuliferum* sp. nov., 89% (differing in 41 nucleotides and 0 indels) from *C. annuliferum* (MN782387–MN782395) and 87–89% (differing in 73 to 87 nucleotides) from *C. crotaloides* (MN710680–MN710699).

The COI gene sequences (ON648861–ON648884) showed high similarity values of 97.1% (differing in 20 to 21 nucleotides and 0 indel) from *Criconema* sp. from Denmark TSH-2020 (MN710767–MN710769) suggesting that they could be the same species. Unfortunately, no morphological data were found for *Criconema* sp. Denmark TSH-2020 and only COI gene sequences were available in GenBank. Species delimitation parameters based on COI supported that both species cannot be separated, although additional morphological data are needed to confirm this status.

All molecular markers studied, except for 18S rRNA, clearly separated the new species from other *Criconema* species. Because two closely related species were detected in the sample from common yew, the few male specimens were confirmed molecularly (100%) to belong to *C. plesioannuliferum* sp. nov.

##### Type Habitat and Locality

*Criconema plesioannuliferum* sp. nov. was found in the rhizosphere of common yew (*Taxus baccata* L.) at Valdepeñas, Jaén province, southern Spain (37°35′32.242″ N, 3°42′38.601″ W).

##### Etymology

The species epithet, *plesioannuliferum*, refers to a compound name from the Greek word *plesios* = near, and *annuliferum*, the closet species of the genus *Criconema*.

##### Type Material

Holotype female (VAL-01), 20 paratypes females, 3 males, and 3 fourth-stage juveniles paratypes (slide numbers VAL-02 to VAL-11) were deposited in the Nematode Collection of the Institute for Sustainable Agriculture, CSIC, Córdoba, Spain; two females at Istituto per la Protezione delle Piante (IPP) of Consiglio Nazionale delle Ricerche (C.N.R.), Sezione di Bari, Bari, Italy (VAL-14); and two females deposited at the USDA Nematode Collection (slide T-7630p).

### 2.4. Distribution of the Criconema annuliferum-Complex

**Figure 12:**
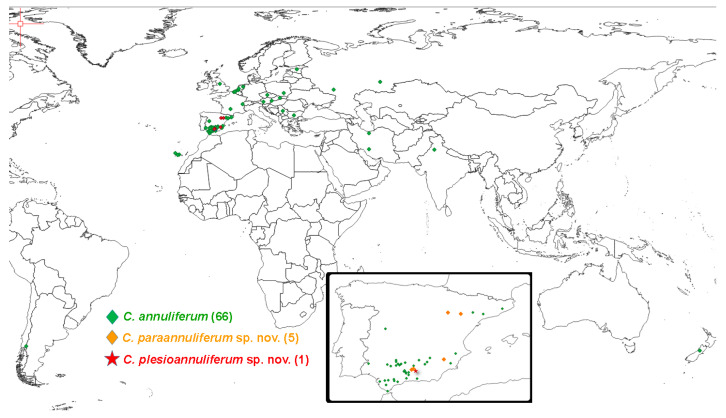
Distribution in the World and Spain of the *Criconema annuliferum*-complex. For each species, the number in brackets indicates the locations cited for each species.

In an exhaustive review of the geographical distribution of the *Criconema annuliferum* -complex in cultivated and natural environments in Spain and all over the world, we detected that this species complex has a wide distribution across a wide variety of herbaceous and woody hosts (Figure 12). The *Criconema annuliferum*-complex is widely distributed in several European countries, but also in Africa [24], Asia [2,25], New Zealand [26], and South America [27]. It should be noted that the highest diversity seems to be in Spain, with three species in cultivated and wild environments (Figure 12). Although these data suggest that other species in this species complex may be found in other countries after accurate integrative taxonomical identifications are conducted on them.

### 2.5. Phylogenetic Analyses of the Criconema annuliferum-Complex

**Figure 13:**
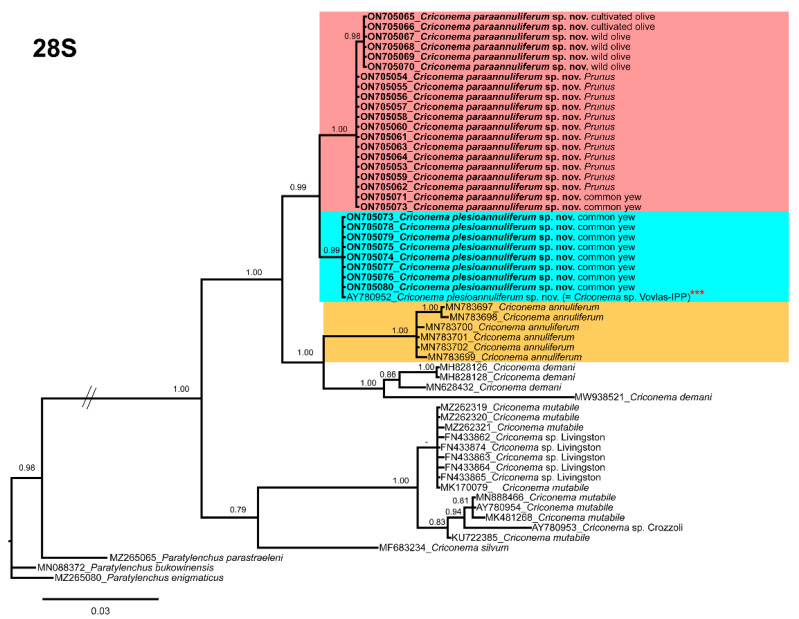
Phylogenetic relationships within the genus *Criconema*. Bayesian 50% majority rule consensus tree as inferred from D2-D3 expansion domains of the 28S rRNA sequence alignment under the general time-reversible model of sequence evolution with a gamma-shaped distribution (GTR + G). Posterior probabilities of more than 0.70 are given for appropriate clades. Newly obtained sequences in this study are in bold. The scale bar indicates expected changes per site, and the coloured boxes indicate the clade association of the *Criconema annuliferum*-complex. *** Initially identified in NCBI as *Criconema* sp. Vovlas-Italy.

The D2–D3 domains of the 28S rRNA gene alignment (702 bp long) included 54 sequences of nine *Criconema* species and three outgroup species (*Paratylenchus bukowinensis* (MN088372), *Paratylenchus enigmaticus* (MZ265080), and *Paratylenchus parastraeleni* (MZ265065). Seventy-eight new sequences were included in this analysis. The Bayesian 50% majority rule consensus tree inferred from the D2-D3 alignment is given in Figure 13. For this region, all species that belong to the species complex *Criconema annuliferum* clustered together in a well-supported (PP = 1.00) clade, which was subdivided into two subclades, one of them (PP = 0.99) formed by *Criconema paraannuliferum* sp. nov. (ON705053–ON705072) and *Criconema plesioannuliferum* sp. nov. (ON705073–ON705080) and the other one (PP = 1.00) by *C. annuliferum* (MN783697–MN783702) and *C. demani* (MH828126, MH828128, MN628432 and MW938521). In this analysis, we detected that an unidentified species of *Criconema* from Italy (AY780952) clustered together with *C. plesioannuliferum* sp. nov., being molecularly identical. Thus, although no morphometrical data on this population are available, it most probably is conspecific with the new species.

**Figure 14:**
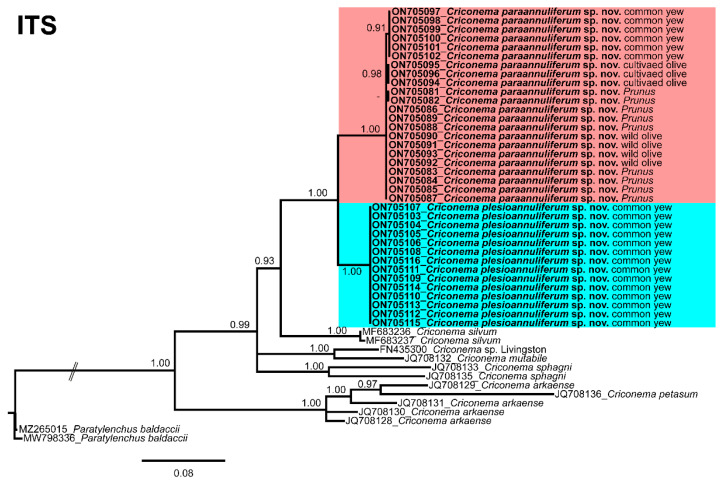
Phylogenetic relationships within the genus *Criconema*. Bayesian 50% majority rule consensus tree as inferred from the ITS rRNA sequence alignment under the transversion model with a gamma-shaped distribution (TVM + G). Posterior probabilities of more than 0.70 are given for appropriate clades. Newly obtained sequences in this study are in bold. The scale bar indicates expected changes per site, and the coloured boxes indicate the clade association of the *Criconema annuliferum*-complex.

The ITS rRNA gene alignment (739 bp long) included 49 sequences of eight species of *Criconema* and two outgroup sequences of *Paratylenchus baldaccii* (MW798336, MZ265015). Thirty-six new sequences were included in this analysis. The Bayesian 50% majority rule consensus tree inferred from the ITS alignment is given in Figure 14. The tree showed a well-supported subclade (PP = 1.00) with *Criconema paraannuliferum* sp. nov. (ON705081–ON705102) and *Criconema plesioannuliferum* sp. nov. (ON705103–ON705116), but clearly separated from each other. 

**Figure 15:**
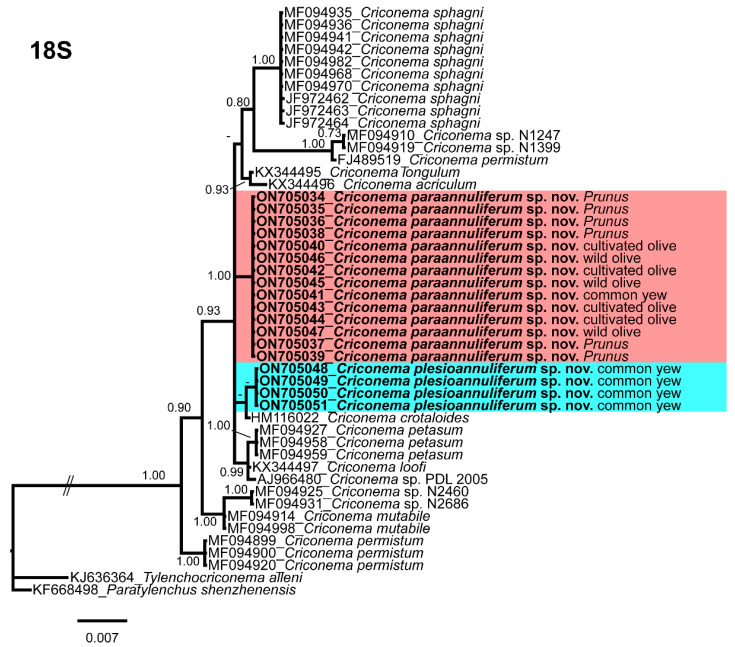
Phylogenetic relationships within the genus *Criconema*. Bayesian 50% majority rule consensus tree as inferred from 18S rRNA sequence alignment under the transition model with invariable sites and a gamma-shaped distribution (TIM2 + I + G). Posterior probabilities of more than 0.70 are given for appropriate clades. Newly obtained sequences in this study are in bold. The scale bar indicates expected changes per site, and the coloured boxes indicate the clade association of the *Criconema annuliferum*-complex.

The 18S rRNA gene alignment (1694 bp long) included 48 sequences of 14 species of *Criconema* and two outgroup species (*Tylenchocriconema alleni* (KJ636364), and *Paratylenchus shenzhenensis* (KF668498)). Twenty-eight new sequences were included in this analysis. The Bayesian 50% majority rule consensus tree inferred from the 18S rRNA sequence alignment is given in Figure 15. The tree showed two well-supported (PP = 1.00) major clades, one of them appears in the basal part of the tree and includes only *C. permistum*, while the other major clade included the remaining species of *Criconema*. For this region, *Criconema plesioannuliferum* sp. nov. clustered into a poorly supported subclade with the other morphologically related species, *C. crotaloides* (HM116022). *Criconema paraannuliferum* sp. nov. formed a unique clade.

**Figure 16:**
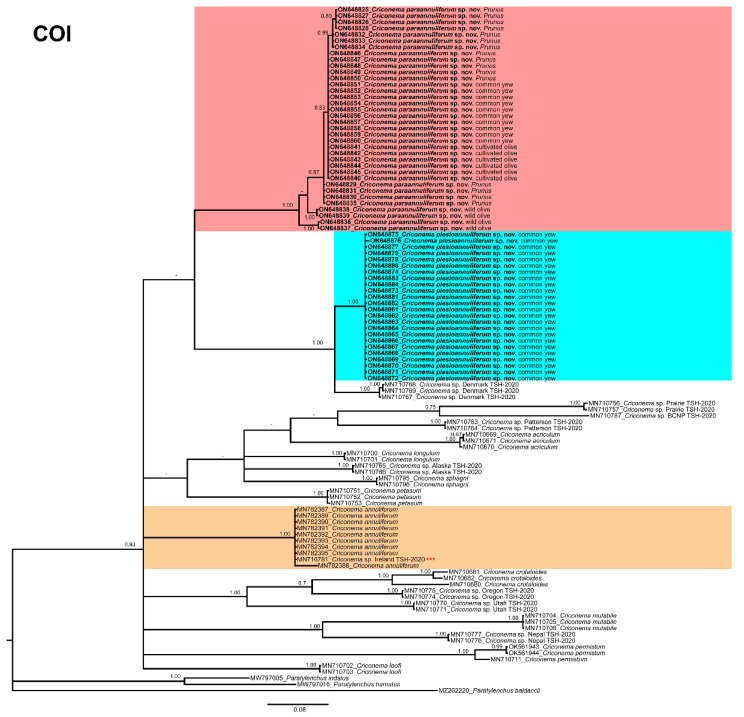
Phylogenetic relationships within the genus *Criconema*. Bayesian 50% majority rule consensus tree as inferred from cytochrome c oxidase subunit 1 (COI) sequence alignment under the three-parameter model with invariable sites and gamma distribution model (TPM3uf + I + G). Posterior probabilities of more than 0.70 are given for appropriate clades. Newly obtained sequences in this study are in bold. The scale bar indicates expected changes per site, and the coloured boxes indicate the clade association of the *Criconema annuliferum*-complex. *** *Criconema* sp. Ireland TSH2020 should be considered to be *C. annuliferum*.

The COI gene alignment (674 bp long) included 110 sequences of 20 species of *Criconema* and three outgroup species (*Paratylenchus baldaccii* (MZ262220), *Paratylenchus hamatus* (MW797016) and *Paratylenchus indalus* (MW797005)). Sixty new sequences were included in this analysis. The Bayesian 50% majority rule consensus tree inferred from the COI sequence alignment is given in Figure 15. The phylogenetic relationships of *Criconema* spp. nov. inferred from analysis of this region were not well defined. The two new species grouped together in a low supported clade with *Criconema* sp. Denmark TSH-2020, which appeared forming a high supported (PP = 1.00) subclade with *C. plesioannuliferum*. The other morphologically related species to this species, *C. crotaloides* and *C. annuliferum*, clustered into two clearly separate well-supported clades (PP = 1.00). An unidentified species of *Criconema* from Ireland (MN710781) clustered within *C. annuliferum* (Figure 16), suggesting that it may be conspecific, but as no morphological data are available, additional studies are needed for confirmation

## 3. Discussion

The primary objective of this study was to identify and molecularly characterize species belonging to the *Criconema annuliferum*-complex associated with cultivated and wild plants (viz. *Prunus* spp., wild and cultivated olive, and common yew) in southern Spain using integrative taxonomical approaches (morphological, morphometrical and molecular). Our results demonstrated that morphological studies integrated with rRNA and mitochondrial DNA molecular markers revealed the cryptic diversity of *Criconema annuliferum* species complex, enabling the description of two new species, *C. paraannuliferum* sp. nov. and *C. plesioannuliferum* sp. nov. Ribosomal and mitochondrial markers (D2-D3 expansion domains of the 28S rRNA gene, ITS rRNA gene, and the mtDNA gene COI) are important tools for accurate identification of *Criconema* spp. In the present study, the mtDNA gene COI was used with others to separate *C. plesioannuliferum* sp. nov. and *C. paraannuliferum* sp. nov. from the Valdepeñas population, as it was the region that showed the highest interspecific variability. The broad range of cultivated and wild hosts indicated that *C. paraannuliferum* sp. nov. is quite generalized and lives in a wide range of environments and host plants. Multivariate morphometric analyses have proven to be useful tools for species delimitation within the genera *Longidorus* and *Xiphinema* [50,51]. Cryptic speciation has commonly been reported in criconematids, consequently these data boosted the hypothesis that criconematid nematodes are a hyper diverse group of organisms [52,53,54]. The recognition of these two new species within the *C. annuliferum*-complex may have a direct influence on the real geographic distribution of these species. The low to moderate nematode population density in the common yew sample, as well as the sequencing of numerous individuals from this site, confirmed that each of the two species represented about half of the sample, suggesting that plant resources are good enough for supporting both species in the same area, and no competitive exclusion is asserted [55]. Notwithstanding this data, additional studies need to be conducted, such as life history phenology to confirm this hypothesis. Interestingly, in some cases, the levels of some of this species (*C. paraannuliferum* sp. nov.) are extremely high (more than 7000 nematodes/500 cm^3^ of soil) in peach and presumably could affect the plant growth. However, phytopathological tests are necessary to understand if these levels are significantly affecting peach production. Nevertheless, the wide distribution and higher molecular diversity of *C. paraannuliferum* sp. nov. in several cultivated and natural environments suggests indirect dispersion by agricultural practices (movement of soil or plant materials) from naturally infested to uninfested soils, although additional analyses on all previous records on *C. annuliferum* from Spain and all over the world can support this hypothesis.

Multivariate morphometric analyses have proven to be useful tools for species delimitation within soil nematodes, especially in plant-parasitic nematodes such as those belonging to the genus *Xiphinema* [56,57,58] and the genus *Longidorus* [51,58]. The present study is, to our knowledge, the first one to apply multivariate methods to delimit and decipher species boundaries within species complexes within the genus *Criconema*. Our data support that the *C. annuliferum*-complex comprises a model example of morphostatic speciation (that is, genetic modifications not reflected in morphology and morphometry) [59], as independent approaches based on molecular analyses using ribosomal and mitochondrial sequence data clearly separated the species considered within the *C. annuliferum*-complex. The results of the multivariate analysis identified the shape and size of the posterior part of the nematode described by the number of annuli between the posterior end of the body and the vulva (RV), the number of annuli on tail (Ran) as well the c’ ratio as key morphometric characters to differentiate some closely related species within the *C. annuliferum*-complex (Table 2, Figure 2). These results agree with the taxonomic statement outlining the number of annuli describing the nematode body as a fundamental feature in identifying the species within the genus *Criconema* [2]. Although some specimens of *C. plesioannuliferum* sp. nov. share similar values for most morphological characters with the other species included in this study, multivariate analysis allowed us to differentiate this species within this cryptic complex using a discrete number of characters (Table 2, Figure 2). However, multivariate analysis also supports the idea that *C. paraannuliferum* sp. nov. and *C. annuliferum* could resemble the same species (Figure 2) based in the wide morphometric variation and the similar values they share for the most diagnostic characters that identify species in the genus *Criconema.* This point could difficult their accurate identification. Finally, our results may also suggest that the *C. annuliferum*-complex comprises an endemic lineage within *Criconema* that has diversified in the Iberian Peninsula. However, further studies exploring the occurrence of the new taxa in other areas are necessary to confirm this hypothesis. 

The lack of intraspecific variability in ribosomal and mitochondrial markers in *C. plesioannuliferum* sp. nov., may suggest a continuous isolation of this population under this natural environment under maintained biological (plant-hosts) and ecological characteristics (soil, temperature, etc.). Alternatively, it may suggest a recent speciation event from *C. paraannuliferum* sp. nov., like that inferred for other criconematids, such as *M. erinaceum* Powers, Mullin, Higgins, Harris & Powers, 2016 [60]. 

Phylogenetic analyses based on the D2-D3 region, ITS, 18S rRNA, and the COI gene using BI, mostly agree with the clustering obtained by other authors [13,16,60,61]. Ribosomal and mitochondrial based phylogenies clearly separate the *C. annuliferum*-complex into three separate species, which was confirmed by morphometric and molecular species delimitation analyses. Unfortunately, a concatenated analysis of the three ribosomal genes was not undertaken due to some sequences not being available for all species.

The present results confirmed previous data describing the remarkable biodiversity of several groups of plant-parasitic nematodes in southern Spain, such as species within the family Longidoridae (including virus vector nematodes of the genera *Xiphinema* and *Longidorus*) or pin nematodes of the genus *Paratylenchus* [51,62,63], and warrant additional sampling efforts to clarify the real biodiversity in our country.

## 4. Materials and Methods

### 4.1. Sampling Sites and Nematode Morphological Identification

A survey was conducted in the five principal areas of stone-fruit production (*Prunus* spp.) in Spain (Almanzora, Ebro, Guadalquivir, Júcar and Segura Valleys), which were established by river valleys. A total of 219 sites were sampled in the present study, and twelve showed the presence of specimens of putative *Criconema annuliferum*. Soil samples for nematode analysis were collected with a shovel from below four to five randomly selected trees and mixed to constitute a soil sample from each sampling site; samples came from the upper 5–40 cm depth of soil. Nematodes were extracted from a 500 cm^3^ sub-sample of soil by centrifugal flotation [64]. In addition, three samples from below common yew and cultivated and wild olives in two localities of Jaen (Valdepeñas and Castillo de Locubín), and one locality from Cádiz province (Prado del Rey), respectively, were selected and studied here, because they contained specimens of *Criconema* closely resembling those from below *Prunus* spp. Nematode identification was performed using an integrative approach, combining morphological and morphometrical evaluation with molecular techniques. Morphological and morphometrical analyses were conducted using fixed individuals mounted on permanent slides. To prepare the fixed material, specimens of *Criconema* were killed at 70–75 °C and fixed in an aqueous solution of 4% formaldehyde + 1% glycerol, dehydrated using alcohol-saturated chamber and processed to pure glycerin using Seinhorst’s method [65] as modified by De Grisse [66]. A total of 94 individuals including 79 females, 8 males and 7 juveniles were used for morphological and morphometrical analyses. Fixed mounted individuals were then examined and measurements of each nematode population, including important diagnosis characteristics (i.e., de Man indices, body length, stylet length, R, Rst, Roes, Rex, RV, Rvan, Ran, and the ratio VL/VB) [41], were performed using a Leica DM6 compound microscope with a Leica DFC7000 T digital camera. 

Females and fourth-stage juveniles of each species mounted in glycerin were selected for SEM observations. The nematodes were hydrated in distilled water, dehydrated in a graded ethanol-acetone series, critical point-dried, coated with gold, and observed with a Zeiss Merlin scanning electron microscope (5 kV) (Zeiss, Oberkochen, Germany) [67].

Voucher specimens of these described species were deposited in the nematode collection of Institute for Sustainable Agriculture, IAS-CSIC, Córdoba, Spain.

### 4.2. DNA Extraction, PCR and Sequencing

For molecular analyses, and to avoid mistakes in case of mixed populations in the same sample, single nematodes were previously mounted in a drop of NaCl and used for molecular identification after recording morphological data. Genomic DNA extraction from single specimens was conducted as described by Palomares-Rius et al. [68]. Briefly, an individual nematode is cut using a scalpel in a drop of PCR buffer (ThermoPol^®^, Biolabs, New England, USA) (20 µL) and 2 μL proteinase K (600 μg/mL) were added. The tubes were frozen at −80 °C (15 min), then incubated at 65 °C (1 h) and at 95 °C (10 min) consecutively. Tubes were centrifuged (1 min, 16,000× *g*) and kept at −20 °C until use in PCR; and more importantly, all three molecular markers for each population of *Criconema* were extracted from the same single individual in each PCR tube without any exception. In addition, male conspecificity was confirmed by single DNA extraction of males for each species.

The D2 and D3 expansion domains of the 28S rRNA were amplified using the D2A (5′-ACAAGTACCGTGAGGGAAAGTTG-3′) and D3B (5′-TCGGAAGGAACCAGCTACTA-3′) primers [69]. The Internal Transcribed Spacer region (ITS) was amplified by using forward primer TW81 (5′-GTTTCCGTAGGTGAACCTGC-3′) and reverse primer AB28 (5′-ATATGCTTAAGTTCAGCGGGT-3′) [70]. The partial 18S rRNA was amplified using the primers 988 (5′-CTCAAAGATTAAGCCATGC-3′) and 1912R (5′-TTTACGGTCAGAACTAGGG-3′) [71]. The COI gene was amplified using the primers COI-F5 (5′-AATWTWGGTGTTGGAACTTCTTGAAC-3′ and COI-R9 (5’-CTTAAAACATAATGRAAATGWGCWACWACATAATAAGTATC-3′) [72]. The PCR cycling conditions for the 28S rRNA, ITS and 18S rRNA were as follows: 95 °C for 15 min, followed by 35 cycles of 94 °C for 30 s, an annealing temperature of 55 °C for 45 s, and 72 °C for 1 min, and one final cycle of 72 °C for 10 min. The PCR cycling for COI primers was as follows: 95 °C for 15 min, 39 cycles at 94 °C for 30 s, 53 °C for 30 s, and 68 °C for 1 min, followed by a final extension at 72 °C for 7 min. The PCR volumes were adapted to 25 µL for each reaction, and primer concentrations were as described in De Ley et al. [69], Subbotin et al. [13], Holterman et al. [71] and Powers et al. [72]. We used 5x HOT FIREpol Blend Master Mix (Solis Biodyne, Tartu, Estonia) in all PCR reactions. The PCR products were purified using ExoSAP-IT (Affimetrix, USB products, Kandel, Germany) and used for direct sequencing in both directions with the corresponding primers. The resulting products were analysed in a DNA multicapillary sequencer (Model 3130XL Genetic Analyzer; Applied Biosystems, Foster City, CA, USA), using the BigDye Terminator Sequencing Kit v.3.1 (Applied Bio-systems) at the Stab Vida sequencing facility (Caparica, Portugal). The sequence chromatograms of the four markers (18S rRNA, ITS, COI and D2-D3 expansion segments of 28S rRNA) were analysed using DNASTAR LASERGENE SeqMan v. 7.1.0. The Basic local alignment search tool (BLAST) at the National Center for Biotechnology Information (NCBI) was used to confirm the species identity of the DNA sequences obtained in this study [73]. The newly obtained sequences were deposited in the GenBank database under accession numbers indicated on the phylogenetic trees and in Table 1.

### 4.3. Recognition of Putative Species within the Criconema annuliferum-Complex and Species Delimitation Approach

Two independent strategies of species delimitation using morphometric and molecular data were used to determine species boundaries within this species complex. The recognition of the group of species included in this complex was established from large-scale taxonomic studies in the genus *Criconema* [2]. Specifically, morphological comparison showed that several of the diagnostic characters defining the genus *Criconema* were characteristic of the whole species complex, highlighting the lip region shape composed by two annuli being the first wider than the second one as well as the total number of annuli on body (R) and between posterior end of body and vulva (RV) [2]. We named the group the *C. annuliferum*-complex, after the oldest described species within it. The main diagnostic features of *C. annuliferum* were used to determine the morphologically closest species to the complex. Thus, in addition to the new taxa, *C. paraannuliferum* sp. nov. and *C. plesioannuliferum* sp. nov., the selected species included in this species complex were *C. crotaloides* and *C. annuliferum* [2]. However, the recognition of the group of species used in both species delimitation approaches as belonging to the *C. annuliferum*-complex (i.e., similar key morphometric characters) was also determined by the presence of accurate studies using an integrative taxonomic strategy to confirm species identity (that is, availability of molecular data linked to accurate morphology identification by morphometry in order to avoid misidentifications). For this reason, the previously described species, *C. crotaloides*, was not included in both strategies because of the lack of available information for it in the literature [17].

Species delineation using morphometry was conducted with exploratory factor analysis (FA) to estimate the degree of association among species within the *C. annuliferum*-complex [74]. Factor analysis was based upon the following morphological characters: L (body length), stylet length, R, Rst, Roes, Rex, RV, Rvan, Ran, and the ratios a, c, c’, V, VL/VB (Table 2) [41]. Several populations from natural and agricultural areas were used for some of the new taxa included in the *C. annuliferum*-complex (Table 1). In the case of the described species *C. annuliferum*, all the nematode populations were selected based on the availability of molecular data, their accurate identification using morphological and morphometrical comparison with other reported populations and closely related species, and/or the species distribution. Thus, the nematode populations selected for *C. annuliferum* were as follows: two populations from Netherlands including the type locality [19,39], a population from Chile [27], two populations from Russia [40], and three populations from Belgium [16]. For these populations, we used the mean value of the morphological characters mentioned above. Overall, 85 female specimens were used in a multivariate approach for the *C. annuliferum*-complex. Prior to the statistical analysis, diagnostic characters were tested for collinearity [75]. We used the collinearity test based on the values of the variance inflation factor (VIF) method that iteratively excludes numeric covariates showing VIF values > 10 as suggested by Montgomery et al. [76]. The FA was performed using the Maximum Likelihood algorithm through a decomposition of the data matrix between populations using the fa function implemented in the software package ‘psych’ [77]. This analysis produced a set of variables (factors) that are linear combinations of the original variables, independent of each other and ranked according to the amount of variation accounted for. Orthogonal varimax raw rotation was used to estimate the factor loadings and only factors with sum of squares (SS) loadings > 1 were extracted. Finally, a minimum spanning tree (MST) based on the Euclidean distance was superimposed on the scatter plot of the *C. annuliferum*-specimens complex against the FA axes. The MST was performed using the ComputeMST function implemented in the software package ‘emstreeR’ [78]. All statistical analyses were performed using the R v. 3.5.1 freeware [79].

Species delineation based on molecular data was performed using the species delimitation plugin [42] from the program Geneious Prime v2022.1.1. (Geneious, Auckland, New Zealand), and was used to calculate intra- and inter-species variation by means of the P ID liberal and the Rosenberg’s P_AB_ value. The intra- and inter-species molecular variation was determined by calculating the ratio between the average genetic distance between individuals within a species and the average genetic distance between individuals belonging to the sister species (the average pairwise tree distance among members of a putative species/the average pairwise tree distance between the members of one putative species and the members of the closest second putative species), if the ratio is less than 0.10 the probability of species identification is high [42]. The P ID (Liberal) value [44] represents the probability that a correct species identification would be made using the best sequence alignment (BLAST), closest genetic distance or placement on a tree (falling within or being sister to a monophyletic species clade). Species with P ID (Liberal) ≥ 0.93 were considered to be adequately delimited [43]. The Rosenberg’s P_AB_ represents the probability that the monophyly of a group of sequences is the result of random branching [45].

### 4.4. Phylogenetic Analyses

The D2-D3 expansion segments of the 28S rRNA, ITS rRNA, 18S rRNA, and COI mtDNA sequences of the seven populations of *Criconema* were obtained in this study. These sequences and other sequences of *Criconema* spp. from GenBank were used for phylogenetic analyses. Selection of outgroup taxa for each dataset were based on previously published studies [16,61]. Multiple sequence alignments of the different genes were completed using the FFT-NS-2 algorithm of MAFFT V.7.450 [80]. The BioEdit program V. 7.2.5 [81] was used for sequence alignments visualization and edited by Gblocks ver. 0.91b [82] using options for a less stringent selection (minimum number of sequences for a conserved or a flanking position: 50% of the number of sequences + 1; maximum number of contiguous non-conserved positions: 8; minimum length of a block: 5; allowed gap positions: with half). The phylogenetic analyses of the sequence datasets were based on Bayesian inference (BI) using MrBayes 3.1.2 [83]. The best-fit model of DNA evolution was achieved using JModelTest V.2.1.7 [84] with the Akaike information criterion (AIC). The best-fit model, the base frequency, the proportion of invariable sites, and the gamma distribution shape parameters and substitution rates in the AIC were then used in MrBayes for the phylogenetic analyses. The general time-reversible model with a gamma-shaped distribution (GTR + G) for the D2-D3 segments of 28S rRNA, the transversion model with a gamma-shaped distribution (TVM + G) for ITS rRNA region, the transition model with invariable sites and a gamma-shaped distribution (TIM2 + I + G) for the partial 18S rRNA gene, and the three-parameter model with invariable sites and gamma distribution model (TPM3uf + I + G) for COI gene, were run with four chains for 4 × 10^6^ generations, respectively. A combined analysis of the three ribosomal genes was not undertaken due to some sequences not being available for all species. The sampling for Markov chains was conducted at intervals of 100 generations. For each analysis, two runs were conducted. After discarding burn-in samples of 30% and evaluating convergence, the remaining samples were retained for more in-depth analyses. The topologies were used to generate a 50% majority-rule consensus tree. On each appropriate clade, posterior probabilities (PP) were given. FigTree software version v.1.4.3 [85] was used for visualizing trees from all analyses.

## 5. Conclusions

This study proves the importance of using integrative taxonomy for the identification of species of *Criconema*. It establishes the existence of cryptic biodiversity within the *C. annuliferum*-complex, increasing and expanding the diversity of this group of nematodes in Spain. For the first time, our results demonstrate the coexistence of two closely related species of *Criconema* within the same sample in a natural environment, suggesting that both species could coexist without competing between themselves (similar individual numbers in the soil sample), while only *C. paraannuliferum* sp. nov. was found in cultivated *Prunus*, probably distributed within cultivated areas by several indirect processes (movements of soils, plant material, etc.). This study provides ribosomal and mitochondrial markers for precise and unequivocal diagnosis of species in the *C. annuliferum*-complex and suggests that other reports of *C. annuliferum* in Spain and around the world need to be confirmed with molecular markers.

## Figures and Tables

**Table 1:** Taxa sampled from the *Criconema annuliferum*-complex in this study from Spain and DNA sequence Genebank accession numbers obtained.

Species	Sample Code	Locality, Province	Host-Plant	D2–D3	ITS	18S	COI
*C. paraannuliferum* sp. nov.	PR-129	Calasparra, Murcia	peach	ON705053–ON705054	ON705081–ON705084	ON705034	ON648825–ON648828
*C. paraannuliferum* sp. nov.	PR-125	Barranda, Murcia	apricot	ON705055	-	ON705035	-
*C. paraannuliferum* sp. nov.	PR-141	Cieza, Murcia	almond	ON705056–ON705057	-	-	-
*C. paraannuliferum* sp. nov.	PR-203	Ricla, Zaragoza	peach	ON705058–ON705059	ON705085–ON705086	ON705036	ON648829–ON648830
*C. paraannuliferum* sp. nov.	PR-217	Quinto de Ebro, Zaragoza	plum	ON705060–ON705061	ON705088–ON705089	ON705037	ON648832–ON648835
*C. paraannuliferum* sp. nov.	PR-201	La Almunia, Zaragoza	almond	ON705062–ON705063	-	ON705038	-
*C. paraannuliferum* sp. nov.	PR-208	Sástago, Zaragoza	apricot	ON705064	ON705087	ON705039	ON648831
*C. paraannuliferum* sp. nov.	NEV-22	Castillo de Locubín, Jaén	cultivated olive	ON705065–ON705066	ON705094–ON705096	ON705040–ON705044	ON648840–ON648845
*C. paraannuliferum* sp. nov.	AR-086	Prado del Rey, Cádiz	wild olive	ON705067–ON705070	ON705090–ON705093	ON705045–ON705047	ON648836–ON648839
*C. paraannuliferum* sp. nov.	VAL-22	Valdepeñas, Jaén	common yew	ON705071–ON705072	ON705097–ON705102	-	ON648846–ON648860
*C. plesioannuliferum* sp. nov.	VAL-22	Valdepeñas, Jaén	common yew	ON705073–ON705080	ON705103–ON705116	ON705048–ON705051	ON648861–ON648884

(“-”) Not obtained or not performed.

**Table 2:** Eigenvector and SS loadings of factors derived from nematode morphometric characters for the *Criconema annuliferum*-complex (*Criconema annuliferum*, *Criconema paraannuliferum* sp. nov., *Criconema plesioannuliferum* sp. nov.).

Character/Ratio ^a,b^	MLC1	MLC2	MLC3
L	−0.29	0.89	−0.11
Stylet length	0.00	0.66	−0.11
R	0.37	0.48	−0.25
RV	0.86	−0.19	0.02
Ran	0.90	−0.23	0.26
a	−0.09	0.54	0.35
b	−0.30	0.61	−0.17
c’	0.73	0.06	0.67
V	−0.12	0.15	−0.53
SS loadings	2.42	2.24	1.04
% of total variance	0.27	0.25	0.12
Cumulative % of total variance	0.27	0.52	0.63

^a^ Based on 8 populations (considering means of each character/ratio) of *Criconema annuliferum*, 57 female specimens of *Criconema paraannuliferum* sp. nov. from paratype population sample and five additional population samples from *Prunus* spp., wild and cultivated olives, and common yew, and 20 female specimens of *Criconema plesioannuliferum* sp. nov. from the paratype population sample of common yew. Values of morphometric variables 1 to 3 (eigenvalue > 0.66) are underlined. All populations were identified molecularly and located in southern Spain. ^b^ Morphological and diagnostic characters according to Hunt and Palomares-Rius [41].

**Table 3:** Parameters evaluating the *Criconema annuliferum*-complex delimitation based on two rRNA genes (D2-D3 region of the 28S rRNA, ITS) and one mtDNA barcoding locus, COI for three *Criconema* species of the complex.

Species	Gene	Intra/Inter ^a^	P ID (Liberal) ^b^	Clade Support ^c^	Rosenberg’s P_AB_ ^d^
*Criconema annuliferum*	D2–D3	0.12	**0.97 (0.87,1.0)** ^e^	**1.00**	**1.06 × 10^−3^**
	ITS	-	-	-	-
	COI	0.02	**1.00 (0.95,1.0)**	**1.00**	**5.3 × 10^−16^**
*Criconema paraannuliferum* sp. nov.	D2–D3	0.13	**0.98 (0.95,1.0)**	**1.00**	**7.1 × 10^−9^**
	ITS	0.02	**1.00 (0.95,1.0)**	**1.00**	**2.4 × 10^−6^**
	COI	0.04	**0.98 (0.93,1.0)**	**1.00**	**6.5 × 10^−0^**
*Criconema plesioannuliferum* sp. nov.	D2–D3	0.09	**0.98 (0.93,1.0)**	**0.99**	**2.2 × 10^−6^**
	ITS	0.02	**1.00 (0.95,1.0)**	**1.00**	**2.4 × 10^−6^**
	COI	0.01	**1.00 (0.98,1.0)**	**1.00**	**2.6 × 10^−5^**

^a^ Intra-species variation relative to inter-species variation. ^b^ The P ID (Liberal) value represents the probability (with the 95%confidence interval) for the prediction, of making a correct identification of an unknown specimen of the focal species using DNA Barcoding (closest genetic distance). P ID (Liberal) values ≥ 0.93 are considered to be delimited [43]. Numbers in bold represent significant values. ^c^ Clade support: posterior probabilities from Bayesian trees. ^d^ Rosenberg’s P_AB_ value is the probability that the monophyly of a group of sequences is the result of random branching. ^e^ Significant results are in bold. (-) Not obtained or not performed because of the lack of ITS for this species in NCBI.

**Table 4:** Morphometrics of *Criconema paraannuliferum* sp. nov. from several localities in Spain.

Locality	Calasparra, Murcia, Peach (PR-129)			Ricla, Zaragoza, Peach (PR-203)	Quinto de Ebro, Zaragoza, Peach (PR-217)	Valdepeñas, Jaén, Common Yew	Castillo de Locubín, Jaén, Cultivated Olive		Prado del Rey, Cádiz, Wild Olive
Character/Ratio ^a^	Holotype	Female Paratypes	Fourth-Stage Juveniles				Females	Males	Females
n	1	20	4	5	5	11	8	5 439.4 ± 31.5 (409–490)	8
L	496	482.1 ± 34.9 (396–537)	333.5 ± 48.5 (305–406)	561.2 ± 61.8 (503–657)	575.6 ± 33.3 (542–624)	508.1 ± 45.4 (446–619)	585.6 ± 18.9 (563–601)		618.2 ± 21.7 (592–651)
R	57	57.8 ± 0.8 (57–59)	61.0 ± 0.8 (60–62)	59.2 ± 1.3 (58–61)	60.4 ± 1.9 (59–63)	60.7 ± 2.6 (57–66)	63.4 ± 1.1 (62–65)	-	63.4 ± 1.1 (62–65)
Rst	12	13.5 ± 1.1 (12–15)	16.0 ± 1.4 (15–18)	13.2 ± 0.8 (12–14)	13.6 ± 0.9 (13–15)	14.1 ± 0.9 (12–15)	13.4 ± 1.1 (12–15)	-	12.8 ± 0.4 (12–13)
Roes	21	18.6 ± 1.8 (15–23)	21.0 ± 2.4 (19–24)	18.8 ± 0.8 (18–20)	18.4 ± 1.1 (17–20)	18.2 ± 1.2 (16–20)	17.6 ± 1.1 (16–19)	-	17.8 ± 1.6 (16–20)
Rex	20	17.8 ± 1.9 (14–22)	20.0 ± 2.4 (18–23)	17.8 ± 0.8 (17–19)	17.6 ± 1.1 (16–19)	19.0 ± 1.4 (17–21)	18.8 ± 1.3 (17–20)	-	18.8 ± 1.6 (17–21)
RV	7	8.5 ± 0.6 (7–10)	-	8.8 ± 0.4 (8–9)	8.8 ± 0.8 (8–10)	9.1 ± 0.5 (8–10)	8.4 ± 0.5 (8–9)	-	8.2 ± 0.8 (7–9)
Rvan	3	4.3 ± 0.7 (3–5)	-	4.4 ± 0.5 (4–5)	4.6 ± 0.5 (4–5)	4.4 ± 0.5 (4–5)	4.2 ± 0.8 (3–5)	-	4.4 ± 0.5 (4–5)
Ran	4	4.2 ± 0.6 (3–6)	4.3 ± 0.5 (4–5)	4.2 ± 0.4 (4–5)	4.2 ± 0.4 (4–5)	4.7 ± 0.5 (4–5)	4.2 ± 0.4 (4–5)	-	4.0 ± 0.7 (3–5)
O	10.6	9.7 ± 1.2 (8.2–13.3)	10.8 ± 2.4 (8.0–13.2)	8.9 ± 0.6 (8.3–9.8)	8.9 ± 0.4 (8.3–9.5)	11.2 ± 3.0 (8.6–14.4)	8.4 ± 0.9 (7.5–9.1)	-	8.5 ± 1.0 (7.8–10.3)
a	11.8	10.1 ± 0.9 (8.6–11.8)	8.3 ± 0.6 (7.6–9.0)	10.3 ± 0.6 (9.7–11.1)	10.8 ± 0.5 (10.4–11.6)	9.4 ± 0.6 (8.7–10.4)	11.5 ± 1.1 (10.2–12.9)	16.3 ± 1.8 (13.9–18.8)	10.7 ± 1.5 (9.2–13.0)
b	3.4	3.5 ± 0.3 (3.0–3.9)	3.0 ± 0.5 (2.6–3.5)	3.9 ± 0.3 (3.5–4.4)	4.1 ± 0.4 (3.8–4.8)	3.6 ± 0.2 (3.2–3.9)	3.9 ± 0.1 (3.7–4.1)	4.5 ± 0.4 (4.0–4.9)	4.0 ± 0.2 (3.6–4.2)
c	24.8	23.7 ± 1.5 (20.3–26.3)	16.6 ± 1.4 (15.3–18.5)	27.2 ± 2.5 (24.0–29.9)	28.3 ± 2.5 (24.6–31.2)	24.4 ± 2.0 (22.2–28.1)	24.3 ± 1.2 (23.1–26.1)	11.1 ± 0.8 (10.3–12.3)	20.7 ± 3.2 (18.4–26.0)
c’	1.1	1.1 ± 0.1 (1.0–1.2)	1.1 ± 0.1 (1.1–1.2)	1.1 ± 0.1 (1.0–1.2)	1.1 ± 0.1 (1.1–1.2)	1.1 ± 0.1 (1.0–1.2)	1.1 ± 0.1 (1.0–1.2)	2.0 ± 0.1 (1.8–2.1)	1.3 ± 0.1 (1.2–1.5)
V or T	88.9	86.9 ± 1.6 (82.8–89.1)	-	87.6 ± 2.0 (84.1–89.1)	87.8 ± 0.6 (87.3–88.7)	87.4 ± 1.5 (84.6–89.8)	87.8 ± 0.5 (87.3–88.6)	-	85.5 ± 1.4 (83.9–86.9)
VL/VB	1.7	1.8 ± 0.1 (1.6–2.0)	-	1.7 ± 0.1 (1.6–1.9)	1.8 ± 0.1 (1.6–1.9)	1.6 ± 0.1 (1.4–1.8)	1.8 ± 0.1 (1.7–1.9)	-	1.7 ± 0.1 (1.5–1.9)
First annulus	15.0	18.0 ± 1.2 (15.0–20.5)	12.6 ± 1.1 (11.5–14.0)	18.0 ± 1.0 (17–19)	19.6 ± 0.9 (19.0–21.0)	18.7 ± 1.3 (16.5–21.0)	19.1 ± 1.7 (17.0–21.0)	-	19.7 ± 1.0 (18.0–20.5)
Second annulus	13.0	15.9 ± 1.0 (13.0–18.0)	12.9 ± 0.9 (12.0–14.0)	16.0 ± 0.7 (15–17)	18.1 ± 0.7 (17.0–19.0)	16.6 ± 1.3 (14.5–19.0)	16.8 ± 2.0 (15.0–19.0)	-	16.6 ± 1.1 (15.0–18.0)
Stylet	90.0	92.5 ± 3.2 (85.0–99.0)	72.1 ± 4.9 (65.0–75.5)	93.6 ± 4.2 (88.0–99.0)	100.2 ± 6.8 (95.0–112.0)	100.8 ± 5.7 (91.0–113.0)	103.7 ± 3.0 (98.5–106.0)	-	106.4 ± 8.1 (92.0–112.0)
conus	77.0	77.8 ± 3.1 (72.0–84.0)	60.1 ± 5.3 (53.0–65.5)	80.0 ± 2.7 (77.0–84.00)	83.4 ± 6.6 (79.0–95.0)	86.5 ± 3.8 (80.0–94.0)	91.0 ± 3.2 (86.0–94.0)	-	92.6 ± 6.1 (82.0–97.0)
Pharynx	148.0	138.0 ± 12.2 (115–168)	111.3 ± 13.6 (91–119)	145.4 ± 3.6 (140–150)	141.8 ± 9.8 (130–157)	142.5 ± 11.8 (132–172)	151.4 ± 5.0 (146–159)	97.8 ± 7.3 (85–102)	155.8 ± 7.4 (148–168)
Max. body width	42.0	47.9 ± 3.0 (42.0–54.0)	40.0 ± 3.6 (37.0–45.0)	54.4 ± 4.5 (48.0–59.0)	53.2 ± 2.8 (50.0–57.0)	54.0 ± 4.8 (45.0–51.0)	51.2 ± 6.1 (44.0–59.0)	27.1 ± 2.3 (24.0–30.0)	58.2 ± 6.3 (50.0–66.0)
Anal body diam.	18.5	18.7 ± 0.5 (17.0–19.0)	17.5 ± 1.3 (16.0–19.0)	18.8 ± 0.4 (18.0–19.0)	18.7 ± 0.4 (18.0–19.0)	19.0 ± 0.7 (18.0–20.0)	22.1 ± 1.2 (23.0–25.0)	19.8 ± 1.3 (18.0–21.0)	22.5 ± 2.3 (19.0–25.0)
Vulva to anus distance	31.0	29.8 ± 1.5 (27.0–32.0)	-	29.6 ± 1.7 (28.0–32.0)	31.0 ± 1.0 (30.0–32.0)	30.3 ± 2.1 (28.0–32.0)	48.0 ± 5.8 (41.0–54.0)	-	35.8 ± 6.0 (28.0–43.0)
Tail	20.0	20.4 ± 0.9 (18.5–22.0)	20.0 ± 1.6 (18.0–22.0)	20.6 ± 1.1 (19.5–22.0)	20.4 ± 0.9 (20.0–22.0)	20.8 ± 1.3 (19.0–23.0)	24.1 ± 0.7 (23.0–25.0)	39.8 ± 2.0 (38.0–43.0)	30.4 ± 4.0 (24.0–34.0)
Spicules	-	-	-	-	-	-	-	33.1 ± 2.8 (30.5–37.0)	-
Gubernaculum	-	-	-	-	-	-	-	12.1 ± 1.8 (9.0–13.5)	-

Measurements are in µm and in the form: (mean) ± (standard deviation), (range). (-) Not obtained or not performed. Abbreviations: a, body length/maximal body width; b, body length/pharyngeal length; c, body length/tail length; c’, tail length/body width at anus; L, (total body length); *n*, number of specimens studied; O, distance between stylet base and orifice of dorsal oesophageal gland as percentage of stylet length; R, total number of body annuli; Roes, number of annuli in pharyngeal region; Rex, number of annuli between anterior end of body and excretory pore; Rst, number of body annuli between labial disc and stylet knobs; RV, number of annuli between posterior end of body and vulva; Rvan, number of annuli between vulva and anus; Ran, number of annuli on tail; V, (distance from anterior end to vulva/body length) × 100; VL/VB, distance between vulva and posterior end of body divided by body width at vulva; T, (distance from cloacal aperture to anterior end of testis/body length) × 100.

**Table 5:** Morphometrics of *Criconema plesioannuliferum* sp. nov. from common yew (*Taxus baccata* L.) in Valdepeñas, Jaén province, Spain.

Character/Ratio ^a,b^	Holotype	Paratypes		
		Females	Males	Fourth-Stage Juveniles
*n*	1	20	3	3
L	489	460.1 ± 65.3 (372–658)	340.7 ± 9.0 (332–350)	302.0 ± 13.7 (287–314)
R	61	61.0 ± 2.6 (57–67)	-	62.3 ± 1.5 (61–64)
Rst	15	14.6 ± 1.4 (12–18)	-	16.7 ± 0.6 (16–17)
Roes	19	20.4 ± 1.0 (19–23)	-	22.3 ± 0.6 (22–23)
Rex	20	20.1 ± 1.5 (17–22)	-	20.3 ± 0.6 (20–21)
RV	11	10.4 ± 0.9 (9–12)	-	-
Rvan	1	1.5 ± 0.5 (1–2)	-	-
Ran	10	8.9 ± 0.8 (8–10)	-	7.0 ± 1.0 (6–8)
O	9.2	10.4 ± 2.1 (8.9–17.0)	-	9.8 ± 0.9 (9.3–10.8)
a	10.4	10.1 ± 1.0 (8.1–12.4)	15.9 ± 0.7 (15.1–16.6)	7.4 ± 0.3 (7.2–7.7)
b	3.6	3.3 ± 0.3 (2.7–4.0)	3.6 ± 0.2 (3.4–3.8)	2.9 ± 0.1 (2.8–2.9)
c	10.4	10.6 ± 1.2 (8.8–13.7)	10.7 ± 1.0 (9.7–11.7)	10.8 ± 0.9 (9.8–11.6)
c’	1.7	1.6 ± 0.1 (1.3–1.8)	2.0 ± 0.2 (1.8–2.3)	1.2 ± 0.1 (1.1-1.3)
V or T	88.5	86.0 ± 1.5 (81.7–88.5)	40.1 ± 4.1 (35.6–43.4)	-
VL/VB	1.8	1.7 ± 0.1 (1.6–2.0)	-	-
First annulus	15.0	16.2 ± 1.3 (14.0–19.0)	-	10.7 ± 0.3 (10.5–11.0)
Second annulus	13.0	14.5 ± 1.1 (13.0–16.5)	-	9.5 ± 0.5 (9.0–10.0)
Stylet	92.0	95.7 ± 5.7 (86.0–108.0)	-	70.2 ± 5.3 (65.0–75.5)
conus	81.0	83.6 ± 4.6 (76.0–92.0)	-	61.5 ± 3.8 (58.0–65.5)
Pharynx	134.0	139.8 ± 26.7 (103–215)	94.0 ± 3.6 (90–97)	105.3 ± 4.0 (101–109)
Max. body width	47.0	45.9 ± 6.8 (36.0–60.5)	21.5 ± 0.9 (20.5–22.0)	41.0 ± 1.0 (40.0–42.0)
Anal body diam.	27.0	27.0 ± 4.8 (22.0–39.0)	15.7 ± 1.5 (14.0–17.0)	23.7 ± 0.6 (23.0–24.0)
Vulva to anus distance	15.0	16.3 ± 2.6 (11.0–21.0)	-	-
Tail	47.0	43.9 ± 8.8 (33.5–66.0)	32.0 ± 3.6 (29.0–36.0)	28.0 ± 2.6 (26.0–31.0)
Spicules	-	-	31.0 ± 1.7 (29.0–32.0)	-
Gubernaculum	-	-	7.0 ± 0.5 (6.5–7.5)	-

Measurements are in µm and in the form: (mean) ± (standard deviation), (range). (-) Not obtained or not performed. Abbreviations: a, body length/maximal body width; b, body length/pharyngeal length; c, body length/tail length; c’, tail length/body width at anus; L, (total body length); *n*, number of specimens studied; O, distance between stylet base and orifice of dorsal oesophageal gland as percentage of stylet length; R, total number of body annuli; Roes, number of annuli in pharyngeal region; Rex, number of annuli between anterior end of body and excretory pore; Rst, number of body annuli between labial disc and stylet knobs; RV, number of annuli between posterior end of body and vulva; Rvan, number of annuli between vulva and anus; Ran, number of annuli on tail; V, (distance from anterior end to vulva/body length) × 100; VL/VB, distance between vulva and posterior end of body divided by body width at vulva; T, (distance from cloacal aperture to anterior end of testis/body length) × 100.

## Data Availability

The datasets generated during and/or analysed during the current study are available from NCBI and from the corresponding author on reasonable request.
